# Otilonium Bromide Exhibits Potent Antifungal Effects by Blocking Ergosterol Plasma Membrane Localization and Triggering Cytotoxic Autophagy in *Candida Albicans*

**DOI:** 10.1002/advs.202406473

**Published:** 2024-07-12

**Authors:** Cheng Zhen, Li Wang, Yanru Feng, Malcolm Whiteway, Sijin Hang, Jinhua Yu, Hui Lu, Yuanying Jiang

**Affiliations:** ^1^ Department of Pharmacy, Shanghai Tenth People's Hospital School of Medicine Tongji University No.1239 Siping Road Shanghai 200092 China; ^2^ Department of Biology Concordia University Montreal QC H4B 1R6 Canada

**Keywords:** autophagy, candida albicans, ergosterol plasma membrane localization, otilonium bromide

## Abstract

Candidiasis, which presents a substantial risk to human well‐being, is frequently treated with azoles. However, drug‐drug interactions caused by azoles inhibiting the human CYP3A4 enzyme, together with increasing resistance of *Candida* species to azoles, represent serious issues with this class of drug, making it imperative to develop innovative antifungal drugs to tackle this growing clinical challenge. A drug repurposing approach is used to examine a library of Food and Drug Administration (FDA)‐approved drugs, ultimately identifying otilonium bromide (OTB) as an exceptionally encouraging antifungal agent. Mechanistically, OTB impairs vesicle‐mediated trafficking by targeting Sec31, thereby impeding the plasma membrane (PM) localization of the ergosterol transporters, such as Sip3. Consequently, OTB obstructs the movement of ergosterol across membranes and triggers cytotoxic autophagy. It is noteworthy that *C. albicans* encounters challenges in developing resistance to OTB because it is not a substrate for drug transporters. This study opens a new door for antifungal therapy, wherein OTB disrupts ergosterol subcellular distribution and induces cytotoxic autophagy. Additionally, it circumvents the hepatotoxicity associated with azole‐mediated liver enzyme inhibition and avoids export‐mediated drug resistance in *C. albicans*.

## Graphical abstract

Otilonium bromide (OTB) demonstrates efficacy against *Candida albicans* by targeting Sec31, thereby impeding vesicle transport and ergosterol localization, ultimately inducing cytotoxic autophagy. *C. albicans* exhibits susceptibility to OTB because it does not act as a substrate of drug pumps. The mechanism of action of OTB, involving modulation of ergosterol distribution and initiation of autophagy, offers a novel antifungal strategy.

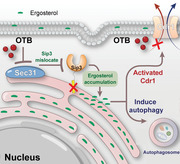

## 1 Introduction

*Candida albicans* can cause a range of diseases, from superficial mucosal and dermal infections to life‐threatening disseminated bloodstream infections, particularly in immunocompromised individuals, and thus pose a serious threat to human health.^[^ ^1^ ^]^ Azoles, widely used for treating candidiasis due to their broad antifungal spectrum, low toxicity, affordability, and versatile administration, target 14‐α‐sterol demethylase to inhibit ergosterol biosynthesis.^[^ ^2^ ^]^ However, a notable obstacle encountered by these drugs, commonly referred to as their “Achilles heel,” is the interaction with the hepatic cytochrome P450 enzyme system. Azoles are metabolized by this system, leading to potential fluctuations in azole levels when interacting with other drugs metabolized by these enzymes. Additionally, azoles can inhibit cytochrome P450 enzymes, which may result in increased plasma concentrations of other drugs metabolized by these enzymes.^[^ ^3^ ^]^ This concern is particularly alarming as many patients with fungal infections also contend with complex medical conditions such as acquired immune deficiency syndrome (AIDS), cancer, and compromised immune function, necessitating multiple drug combinations. Given that the transport and integration of ergosterol into the plasma membrane (PM) are crucial processes for its optimal function,^[^ ^4^ ^]^ impeding its proper distribution may mimic the inhibition of ergosterol synthesis, potentially circumventing the drug‐drug interactions caused by azoles inhibiting the CYP3A4 enzyme. However, there is currently a dearth of compounds that interfere with ergosterol transportation to the PM while demonstrating antifungal properties.

A further important challenge for azoles lies in the capability of drug efflux pumps to expel these compounds from *C. albicans* cells, allowing routes to the development of azole resistance.^[^ ^5^ ^]^ This situation occurs because, on the one hand, azoles induce the expressions of the efflux pumps, such as Cdr1, Cdr2, and Mdr1,^[^ ^6^ ^]^ while on the other hand, azoles themselves are substrates of the efflux pumps.^[^ ^7^ ^]^ Consequently, the over‐expressed efflux pumps induce the efflux of azoles, rendering *C. albicans* tolerant to these compounds and potentially fostering azole resistance.^[^ ^8^ ^]^ Recently, the compound iKIX1 has been found to disrupt the interaction between the mediator complex and Pdr1, a transcriptional regulator responsible for the expression of the efflux pump Pdr5. This disruption effectively inhibits the expression of the pump and enhances the efficacy of FLC against FLC‐resistant Candida glabrata.^[^ ^9^ ^]^ Currently, the major mechanisms of azole resistance in pathogenic fungi are ineffective inhibition of the target of azoles and increased efflux. Therefore, a promising and effective antifungal approach involves identifying an antifungal agent that disrupts ergosterol function without being a substrate for drug pumps.

Autophagy, a key physiological mechanism, plays a crucial role in maintaining cellular homeostasis by facilitating the degradation of damaged cytoplasm, proteins, and organelles through transport to lysosomes or vacuoles.^[^ ^10^ ^]^ Various stressful conditions, including nutritional deprivation, metabolic damage, oxidative stress, pathogens, DNA damage, and proteotoxic cues, have been found to upregulate autophagy.^[^ ^10^ ^]^ Autophagy is intricately linked to cellular protection and cell death, with numerous studies demonstrating its dual functions in eukaryotic cells: autophagy activation can either promote cell survival (protective autophagy) or contribute to cell death (cytotoxic autophagy).^[^ ^11^ ^]^ Research has consistently highlighted the key role of autophagy in influencing the growth, development, and virulence factor formation of fungal cells, thus closely correlating with fungal pathogenicity.^[^ ^12^ ^]^ Notably, azoles have been observed to induce endoplasmic reticulum (ER) stress, triggering ER phagy‐like autophagy in *C. albicans* and enhancing autophagic flux.^[^ ^13^ ^]^ Additionally, rapamycin, a well‐known autophagy inducer, demonstrates clear antifungal activity.^[^ ^14^ ^]^ These findings suggest that activating autophagy could represent a promising avenue for antifungal therapy, underscoring the need for the development of novel and more potent autophagy inducers for the effective treatment of candidiasis.

This study employed a drug repositioning strategy to conduct a high‐throughput library screen of compounds approved by the Food and Drug Administration (FDA). Through this screening process, otilonium bromide (OTB) was identified as a highly effective antifungal agent, displaying a low minimum inhibitory concentration (MIC) value, a broad spectrum of antifungal activity, and negligible cytotoxicity. In addition, OTB exhibited fungistatic properties while inhibiting filamentation and biofilm formation in *C. albicans*. Our findings indicate that OTB impaired vesicle‐mediated trafficking by targeting Sec31, thereby impeding the PM localization of ergosterol transporters such as Sip3. As a result, OTB disrupted the transport of ergosterol across membranes and induced cytotoxic autophagy. Notably, OTB was not transported by drug pumps, thus bypassing a typical mechanism of *C. albicans* resistance. This research identified a novel antifungal strategy wherein OTB effectively impedes the transfer of ergosterol between membranes and triggers cytotoxic autophagy.

## 2 Results

### 2.1 Identification of OTB Acting as an Antifungal Agent In Vitro and In Vivo

**Figure 1:**
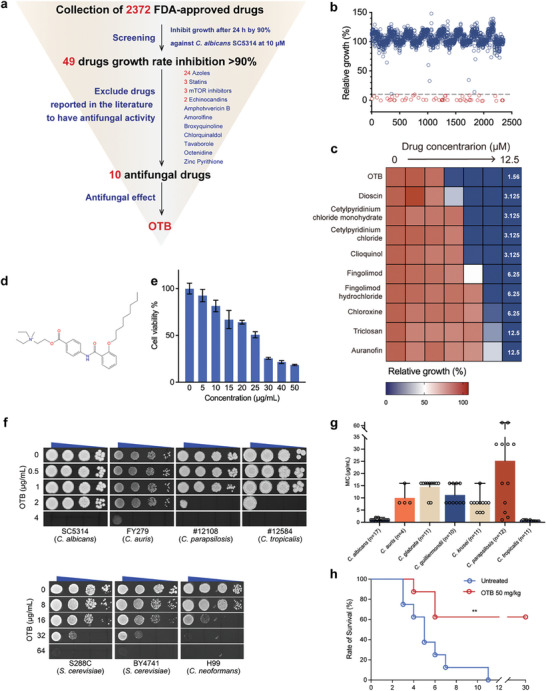
Identification of OTB acting as an antifungal agent in vitro and in vivo. a) Schematic representation of the screening process. b) Screening data are reported as the percent of growth inhibition (%) in the presence of FDA‐approved drugs (10 µM). Compounds are color‐coded based on their significance in inhibiting fungal growth (red, ≥ 90% growth inhibition) or show no significance (blue, < 10% growth inhibition). c) Broth microdilution assays of the top 10 compounds identified in the primary screen. Technical duplicates were averaged, and measurements were normalized to no‐drug controls. Color quantitatively represents relative growth (see scale bar at bottom). MIC values of the compounds were determined as the minimum compound concentration with an inhibition rate exceeding 50%. d) Structure of OTB. e) Cytotoxicity of OTB assessed by CCK‐8 tests on human intestinal epithelial cells (Caco‐2). Bars represent the mean ± SD of three biological replicates. f) Spot assays of OTB against *C. albicans* (SC5314), *C. auris* (FY279), *C. parapsilosis* (#12 108), *C. tropicalis* (#12 584), *S. cerevisiae* (S288C, BY4741), and *C. neoformans* (H99). g) Broth microdilution assays with OTB against various *Candida* species (n = 17 for *C. albicans*, n = 4 for *C. auris*, n = 11 for *C. glabrata*, n = 10 for *C. guilliermondii*, n = 11 for *C. krusei*, n = 12 for *C. parapsilosis*, and n = 11 for *C. tropicalis*. MIC values were determined as described in (c). h) Antifungal activity of OTB against *C. albicans* (SN152) in vivo. Survival curves depict the outcomes of C57BL/6 mice infected with SN152 in a model of *C. albicans* infection in the gastrointestinal tract, treated with OTB at a dose of 50 mg kg^−1^. Survival was plotted as a Kaplan–Meier survival curve. Significance was determined by the Log‐rank test. *p*‐value: **<0.01. *p*‐value, compared to untreated: 0.0078.

A high‐throughput screen of an FDA‐approved drug library of 2372 drugs was conducted to identify new antifungal agents. The antifungal activity of each of the 2372 drugs at a concentration of 10 µM was tested on *C. albicans* SC5314. Forty‐nine drugs were identified as having clear antifungal activity, as they could inhibit *C. albicans* growth by more than 90% compared to the control group without drugs (**Figure** **1**a,b). Subsequently, a total of 39 of the 49 drugs were excluded from further investigation due to their known antifungal activity, including azoles (n = 24), statins (n = 3), the target of rapamycin inhibitors (n = 3), echinocandins (n = 2), amphotericin B (AmB), amorolfine, broxyquinoline, chlorquinadol, octenidine, tavaborole, and zinc pyrithione (Figure 1a).^[^ ^15^ ^]^ The remaining 10 drugs were subjected to evaluation of their antifungal activity by measuring their MIC values against *C. albicans*. Among these drugs, auranofin, triclosan, chloroxine, fingolimod, and fingolimod hydrochloride exhibited antifungal activity against *C. albicans* only at high concentrations, with MIC values of 12.5 µM, 12.5 µM, 6.25 µM, 6.25 µM, and 6.25 µM, respectively (Figure 1c). Dioscin showed antifungal activity with a MIC value of 3.125 µM, but it can cause apoptosis and necrosis in liver cells at concentrations as low as 0.5 µM.^[^ ^16^ ^]^ Cetylpyridinium chloride monohydrate and cetylpyridinium chloride are currently limited to roles as antibacterial mouthwashes because they have shown toxicity in mice, rats, and rabbits and can cause severe eye irritation.^[^ ^17^ ^]^ Clioquinol has been withdrawn from the market due to reports of neurotoxicity in Japanese patients.^[^ ^18^ ^]^ OTB (Figure 1d), however, is commonly administered orally to treat patients with irritable bowel syndrome.^[^ ^19^ ^]^ It exhibited the lowest MIC value (1.56 µM, ≈0.88 µg mL^−1^) against *C. albicans*, indicating its greater antifungal activity compared to the other investigated drugs (Figure 1c). OTB also demonstrated minimal cytotoxicity against human intestinal epithelial Caco‐2 cells with a half‐maximal inhibitory concentration (IC_50_) value of 22.54 ± 1.78 µg mL^−1^ (Figure 1e). This IC_50_ value surpasses the MIC value of OTB against *C. albicans*, suggesting that OTB held promise in terms of safety for the therapeutic management of candidiasis.

Our spot assay results indicate that OTB exhibited potent antifungal activity against various other fungal species, including non‐*albicans Candida* species (Candida auris, Candida parapsilosis, and Candida tropicalis), the yeast *Saccharomyces cerevisiae*, and the basidiomycete *Cryptococcus neoformans* (Figure 1f). Furthermore, the strong antifungal efficacy of OTB was consistently observed across different clinical isolates, such as *C. albicans* (n = 17) with MIC values ranging from 0.5 to 2 µg mL^−1^, *C. auris* (n = 4) with MIC values ranging from 8 to 16 µg mL^−1^, *C. glabrata* (n = 11) with MIC values ranging from 8 to 16 µg mL^−1^, *C. guilliermondii* (n = 10) with MIC values ranging from 8 to 16 µg mL^−1^, *C. krusei* (n = 11) with MIC values ranging from 4 to 16 µg mL^−1^, *C. parapsilosis* (n = 12) with MIC values ranging from 4 to16 µg mL^−1^, and *C. tropicalis* (n = 11) with MIC values ranging from 0.5 to 1 µg mL^−1^ (Figure 1g). These findings demonstrate that OTB exhibited broad‐spectrum antifungal properties against clinical isolates of *Candida* species.

Subsequently, the efficacy of OTB in the treatment of invasive candidiasis was evaluated using a mouse model of the disease, where *C. albicans* is administered via the intestines. Compared to the control group, OTB (50 mg kg^−1^) significantly increased the median survival time from 5 days to 30 days and improved the survival rate from 0% to 62.5% (*p* = 0.0075, log‐rank) (Figure 1h). Overall, OTB demonstrated potent antifungal activity with a low MIC, a wide range of antifungal efficacy, and minimal cytotoxic effects against Caco‐2 cells.

### 2.2 OTB Acted as a Fungistatic Agent and Inhibited Filamentation and Biofilm Formation of *C. Albicans*

**Figure 2:**
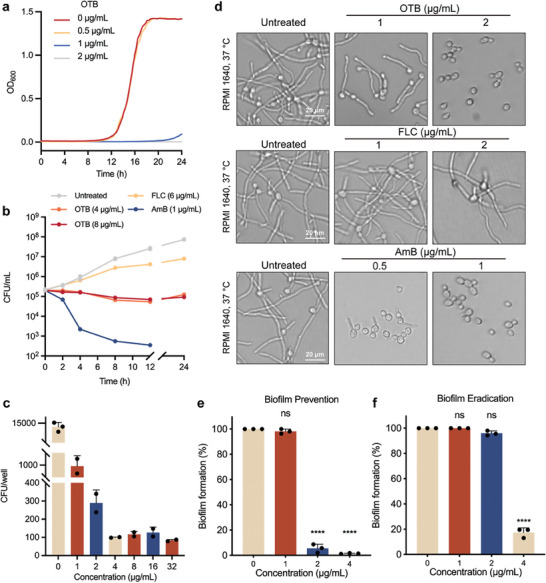
OTB acts as a fungistatic agent and inhibits filamentation and biofilm formation of *C. albicans*. a) For growth inhibition curve assays, *C. albicans* (SN152) cells were treated with OTB (0.5, 1, or 2 µg mL^−1^) or DMSO (control) in a fresh YPD medium. Optical density was measured every 15 min for 48 h. b) For time‐kill curve assays, *C. albicans* (SN152) cells were treated with OTB (4 or 8 µg mL^−1^), FLC (6 µg mL^−1^), AmB (1 µg mL^−1^), or DMSO (control). Triplicate‐sample aliquots were plated at every time point. c) For the MFC assay, *C. albicans* (SN152) cells were treated with OTB (1‐32 µg mL^−1^). After treatment, triplicate‐sample aliquots were plated on drug‐free SDA plates. d) Cultures were grown under filament‐inducing conditions with the indicated compound treatment. Scale bar, 20 µm. Images are representative of three independent biological replicates. e) The preventive effect of OTB against biofilm formation. Data are presented as mean ± SD of technical triplicates, Significance was determined by one‐way ANOVA with Bonferroni's multiple comparisons test; *p*‐value: ****<0.0001. f) The eradicating effect of OTB against mature biofilm. Data are presented as mean ± SD of technical triplicates. Significance was determined by one‐way ANOVA with Bonferroni's multiple comparisons test; *p*‐value: ****<0.0001.

The growth inhibition assays demonstrated that OTB (1 µg mL^−1^) caused a potent inhibitory effect on *C. albicans* (**Figure** **2**a). However, when *C. albicans* cells were exposed to OTB at a concentration of 8 µg mL^−1^ for more than 48 h, no fungicidal effect was observed in the time‐kill curve assays (Figure 2b). Furthermore, the minimum fungicidal concentration (MFC) was not detected even at a concentration of 32 µg mL^−1^. Consequently, the MFC to MIC ratio of OTB exceeded 32 (Figure 2c), indicating fungistatic behavior (MFC: MIC > 4).^[^ ^20^ ^]^

Filamentation helps *C. albicans* penetrate the host tissues, with subsequent invasive growth leading to systemic infection.^[^ ^21^ ^]^ The yeast‐to‐hyphae transition is also critical for biofilm biogenesis, strengthening and supporting the development of heterogeneous biofilm structures.^[^ ^22^ ^]^ In the present study, we observed that treatment of *C. albicans* with low concentration (1 µg mL^−1^) OTB effectively inhibited the morphological transition from yeast to hyphae, and at 2 µg mL^−1^, OTB completely blocked this transition (Figure 2d). Due to the inhibitory effect of OTB on hyphal growth, we tested its inhibitory impact on the formation of biofilms and its effect on mature biofilms using the XTT (sodium 3′‐[1‐(phenylamino‐carbonyl)−3,4‐tetrazolium]‐bis [4‐methoxy‐6‐nitro] benzene sulfonic acid hydrate) reduction assay. During the early phase (immediately after adhesion), biofilm formation in the 2 µg mL^−1^ OTB group was less than 90% compared with the control group (*p* = 0.000013) (Figure 2e). Treatment with 4 µg mL^−1^ of OTB at the biofilm maturation stage decreased biofilm formation by more than 80% (*p* = 0.000033), which represented an efficacy greater than that of FLC (Figure 2f).

### 2.3 The Antifungal Activity of OTB Depended on Ergosterol

OTB has been found to bind to L‐type voltage‐gated calcium channels in the smooth muscle cells of the gut, resulting in the inhibition of calcium ion (Ca^2+^) influx into the cells and a subsequent reduction in acetylcholine release from nerve terminals.^[^ ^19^ ^]^ Thus, we speculated that OTB might act as an antifungal agent by disrupting Ca^2+^ homeostasis in *C. albicans*. However, we found that the addition of Ca^2+^ or the depletion of Ca^2+^ using the Ca^2+^ chelator ethylene glycol tetraacetic acid (EGTA) did not affect the antifungal activity of OTB (Figure S1a, Supporting Information). Similarly, the loss of a catalytic subunit of calcineurin (encoded by the *CMP1*gene) and its downstream transcriptional factor Crz1 (encoded by the *CRZ1* gene), both important for maintaining *C. albicans* Ca^2+^ homeostasis, did not affect the antifungal activity of OTB against *C. albicans* (Figure S1b, Supporting Information). These results suggest that the mechanism by which OTB acts as an antifungal agent does not involve disrupting Ca^2+^ homeostasis.

In general, the positively charged head group in quaternary amine compounds can be attracted by the negative charge on the microorganism's surface to form an electrostatic bond, which increases the pressure on the cell wall and promotes the penetration of long‐chain alkyl moieties into the cell membrane and eventually leads to the leakage of intracellular contents and cell death.^[^ ^23^ ^]^ Cell leakage can be assessed by measuring intracellular components released to the medium to evaluate cell permeability. Light absorption at 260 nm indicates nucleotides, while the absorption at 280 nm indicates protein release.^[^ ^24^ ^]^ The addition of AmB at a concentration of 0.125 µg mL^−1^ significantly increased the absorption values at 260 nm and 280 nm in the culture supernatant of *C. albicans*, suggesting that AmB disrupted the integrity of the PM. In contrast, a high concentration of OTB (4 µg mL^−1^, four times the MIC value of OTB) did not influence the absorption values at 260 nm and 280 nm in the culture supernatant, indicating that OTB did not alter the permeability of the PM in *C. albicans* (Figure S1c, Supporting Information). Our findings indicate that, contrary to the positive control group treated with AmB, OTB treatment did not elicit any discernible effect on membrane potential (Figure S1d, Supporting Information). Additionally, it was observed that the presence of the representative functional membrane lipid components phosphatidylcholine (PC) and phosphatidylethanolamine (PE) did not hinder the antifungal activity of OTB. However, a notable decrease in the antifungal efficacy of octenidine, an antiseptic agent and cationic surfactant known to interact with bacterial cell membranes,^[^ ^25^ ^]^ was observed (Figure S1e, Supporting Information). Moreover, the interaction between OTB and the membrane disruptor AmB exhibited indifference, as evidenced by the fractional inhibitory concentration index (FICI) of these two drugs, which is 2 (Figure S1f, Supporting Information). These findings suggest that the antifungal efficacy of OTB does not rely on direct cell membrane destruction.

**Figure 3:**
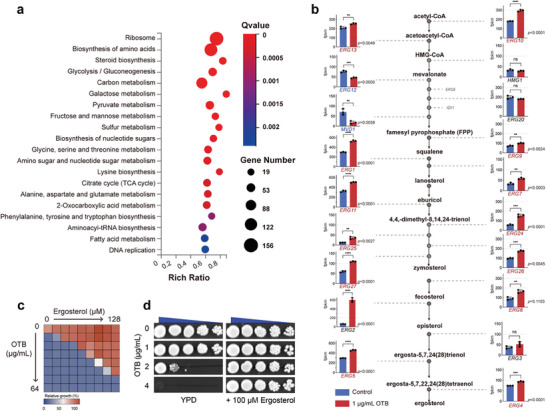
The antifungal activity of OTB is contingent upon the dysfunction of ergosterol. a) RNA‐Seq analysis of cells treated with OTB. KEGG analysis revealed that OTB primarily impacts biological processes related to the ribosome, amino acid biosynthesis, and steroid biosynthesis, based on RNA‐seq analysis of *C. albicans* (SC5314) cells treated with OTB (1 µg mL^−1^). b) Analysis of the transcriptional expression of genes contributing to ergosterol biosynthesis in the RNA‐seq results. Significance was determined by one‐way ANOVA with Bonferroni's multiple comparisons test. *p*‐value: **<0.01, ***<0.001, ****<0.0001. c) Dose‐matrix titration assays with ergosterol in combination with OTB. Color quantitatively represents relative growth (see bar for scale at bottom). d) Spot assays of OTB against *C. albicans* (SN152) in the absence and presence of 100 µM ergosterol.

To investigate the mechanism by which OTB acts against fungi, we carried out an RNA‐seq assay. The RNA‐seq analysis revealed that the genes exhibiting altered expression levels in response to OTB were predominantly associated with steroid biosynthesis (**Figure** **3**a). Subsequent analysis demonstrated that OTB can induce the expression of genes involved in ergosterol biosynthesis, including the *ERG1, ERG2, ERG4, ERG5, ERG6, ERG7, ERG9, ERG10, ERG11, ERG12, ERG13, ERG24, ERG25, ERG26*, and *ERG27* genes (Figure 3b). When *C. albicans* is in a state of ergosterol deficiency, the ergosterol synthesis pathway will be activated to cope with this situation.^[^ ^26^ ^]^ Therefore, we speculated that OTB disrupts intracellular ergosterol in *C. albican*s. This speculation is supported by the observation that the antifungal activity of OTB was neutralized when exogenous ergosterol was introduced, as demonstrated by dose‐matrix titration and spot assays (Figure 3c,d).

### 2.4 OTB Blocked the PM Localization of Ergosterol

Because the antifungal effect of OTB depends on ergosterol, we postulated that OTB could function as an antifungal agent by inhibiting ergosterol biosynthesis. This would predict that OTB would augment the antifungal efficacy of known ergosterol inhibitors. However, the addition of OTB did not have any impact on the antifungal activity of such ergosterol inhibitors, such as fluvastatin (targeting Hmg1, FICI = 3, indifferent), terbinafine (targeting Erg1, FICI = 3, indifferent), and FLC (targeting Erg11, FICI = 3, indifferent) (Figure S2a, Supporting Information). Furthermore, deleting the transcriptional regulator Upc2, which plays a crucial role in the response to ergosterol depletion stress,^[^ ^27^ ^]^ greatly increased the susceptibility of *C. albicans* to ergosterol inhibitors, such as FLC.^[^ ^28^ ^]^ However, OTB exhibited similar antifungal activity against both the *upc2*Δ/Δ null mutant and the wild‐type strain (Figure S2b,c, Supporting Information). This suggests that OTB did not have a significant impact on reducing the intracellular ergosterol content. Furthermore, we examined the effects of ergosterol biosynthesis‐related genes on the antifungal activity of OTB. Our findings indicate that, compared to the wild‐type strain, except for a minor enhancement in the susceptibility of the *erg4*Δ/Δ null mutant to OTB, there were no alterations in the susceptibility of the other null mutants (*erg1*Δ/Δ, *erg2*Δ/Δ, *erg3*Δ/Δ, *erg5*Δ/Δ, *erg6*Δ/Δ, *erg24*Δ/Δ, *erg25*Δ/Δ, and *erg251*Δ/Δ) to OTB (Figure S2d, Supporting Information). Moreover, in contrast to FLC, which generated significant inhibition of ergosterol synthesis, OTB demonstrated minimal inhibitory effects on ergosterol biosynthesis (Figure S2e, Supporting Information). These findings collectively indicate that OTB did not function as an inhibitor of ergosterol synthesis.

**Figure 4:**
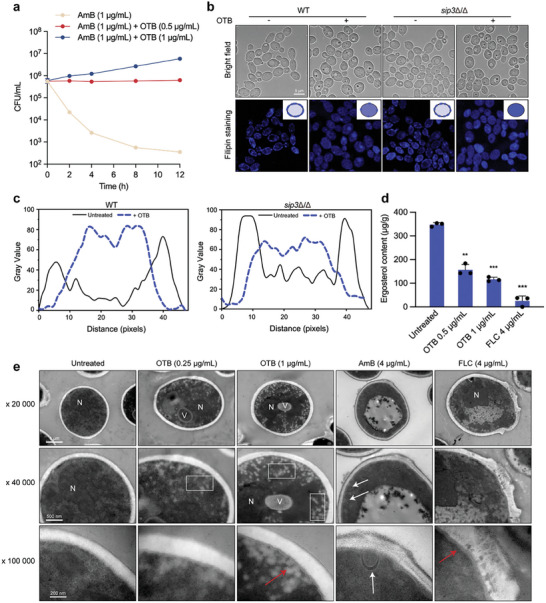
OTB induces a reduction in ergosterol on the *C. albicans* membrane, leading to intracellular membrane invagination. a) For Time‐kill curve assays, *C. albicans* (SN152) cells were treated with OTB (0.5 or 1 µg mL^−1^) before treating with AmB (1 µg mL^−1^) or DMSO (control). Triplicate‐sample aliquots were plated at every time point. b) Ergosterol was visualized by confocal microscopy of cells stained with filipin after treatment with OTB. Assays were performed in biological triplicate. Scale bar, 10 µm. c) The fluorescence intensity distribution curves of the representative single cells in (c). d) The content of plasma membrane ergosterol (µg g^−1^ dry mass of isolated lipids, means ± SD, n = 3) of *C. albicans* (SN152) after OTB (0.5 or 1 µg mL^−1^) or FLC (4 µg mL^−1^) treatment relative to internal cholesterol standard. Data are presented as mean ± SD of triplicate runs. Significance was determined by one‐way ANOVA with Bonferroni's multiple comparisons test; p‐value: *<0.05, **<0.01, ***<0.001. e) Transmission electron micrographs of cells treated with OTB (0.25 or 1 µg mL^−1^), AmB (4 µg mL^−1^), and FLC (4 µg mL^−1^). Red arrows indicate cell membrane shrinkage. White arrows indicate cell membrane rupture. Boxes indicate the demonstration of disorganization of the cytoplasm and the generation of many small vesicles. N, nucleus; V, vacuole; The experiment was repeated in three biologically independent replicates with similar results.

Given that OTB functioned as an antifungal agent depending on ergosterol, but it was not an ergosterol synthesis inhibitor, we speculated that OTB may block membrane transport of ergosterol. If so, the amount of ergosterol localized to the PM would decrease, thereby increasing the resistance of *C. albicans* to AmB because AmB exerts its antifungal activity by directly binding to ergosterol in the PM.^[^ ^29^ ^]^ To investigate this inference, we pretreated *C. albicans* cells without or with OTB, followed by testing the antifungal activity of AmB. The results show that OTB counteracted the fungicidal effect of AmB (**Figure** **4**a), suggesting that OTB reduced the amount of ergosterol in the PM.

We further probed the effect of OTB on the distribution of ergosterol in *C. albicans* cells by staining ergosterol with filipin.^[^ ^30^ ^]^ Compared to the control, the cells treated with 1 µg mL^−1^ OTB showed weaker filipin fluorescence at the PM, while intracellular fluorescence increased, suggesting a decrease in ergosterol at the PM after the OTB treatment of cells (Figure 4b,c). Sip3 is an ergosterol transfer protein that has a role in the transport of ergosterol from the ER to the PM.^[^ ^31^ ^]^ By performing filipin staining on the *sip3*Δ/Δ null mutant, we found that the *sip3*Δ/Δ null mutant showed discontinuous fluorescence at the PM, suggesting that the *sip3*Δ/Δ null mutant had abnormal ergosterol distribution (Figure 4b,c). The fluorescence at the PM in the *sip3*Δ/Δ null mutant is increasingly diffused into the cytoplasm after the treatment of 1 µg mL^−1^ OTB (Figure 4b,c). Furthermore, we extracted the PM from *C. albicans* and quantified the impact of OTB on ergosterol levels in the PM. Our results indicate that OTB successfully decreases the ergosterol content in the PM (Figure 4d).

Since ergosterol is crucial for maintaining the integrity and fluidity of the membrane, we performed transmission electron microscopy of *C. albicans* treated with different concentrations of OTB, AmB, or FLC. When *C. albicans* cells were exposed to 4 µg mL^−1^ FLC, the PM of *C. albicans* cells exhibited extensive irregularities and formed membrane vesicles in the proximity of the PM (Figure 4e). AmB (4 µg mL^−1^) disrupted the integrity of the PM of *C. albicans* cells (white arrows), resulting in the formation of a large vacuole in the center of the cell and the cytosolic content being largely released due to osmotic instability (Figure 4e). In contrast, *C. albicans* treated with OTB (0.25 µg mL^−1^ and 1 µg mL^−1^) had a relatively normal cell structure and cytoplasmic homeostasis, but there was shrinkage of the PM (red arrows), and we observed many small vacuoles located inside the cell and close to the cell wall (boxes) (Figure 4e). In summary, OTB appeared to block the PM localization of ergosterol in *C. albicans*.

### 2.5 OTB Hindered Sip3‐Mediated PM Localization of Ergosterol

**Figure 5:**
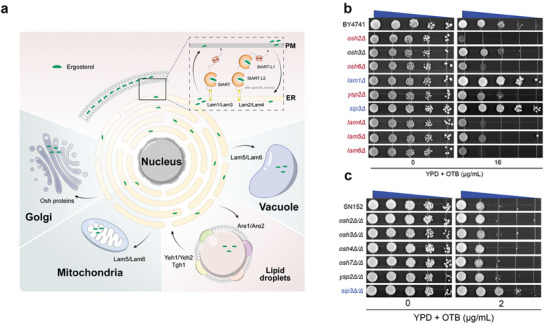
OTB blocks the membrane‐to‐membrane transport of ergosterol. a) The ergosterol transport process within *C. albicans*. b) Spot assays of OTB were performed against wild‐type BY4741 and null mutant strains of *OSH2*, *OSH3*, *OSH6*, *LAM1*, *YSP2*, *SIP3*, *LAM4*, *LAM5*, and *LAM6*. c) Spot assays of OTB were performed against wild‐type SN152 and null mutant strains of *OSH2*, *OSH3*, *OSH4*, *OSH7*, *YSP2*, and *SIP3*.

After being synthesized in the ER, ergosterol is primarily transported to different cellular membranes, particularly the PM.^[^ ^32^ ^]^ The movement of sterols between the ER and the PM predominantly occurs through non‐vesicular mechanisms facilitated by LTPs anchored at membrane contact sites (Lam) and oxysterol‐binding homology (Osh) proteins.^[^ ^4c^ ^]^ The Osh proteins, found in the cytosol, have been demonstrated to bind and transport diverse lipid species, such as sterols, anionic phospholipids, and phosphoinositides.^[^ ^33^ ^]^ Osh3, Osh4, and Osh5 have been identified as mediators of sterol transport from the PM to the ER.^[^ ^4c^ ^]^ In contrast to the Osh proteins, which are distributed throughout the cytoplasm, Lam lipid transfer proteins are anchored specifically at membrane contacts. In *S. cerevisiae*, there are three pairs of paralogous Lam proteins: Lam1 (Ysp1)/Lam3 (Sip3), Lam2 (Ysp2)/Lam4, and Lam5/Lam6.^[^ ^34^ ^]^ The paralogs Lam1/Lam3 and Lam2/Lam4 are found in the contact sites between the ER and the PM, whereas the localization of Lam5/Lam6 is not near the PM (**Figure** **5**a).^[^ ^29^ ^]^

To detect the mechanism of the inhibitory effect of OTB on the PM localization of ergosterol, we tested the susceptibility of *S. cerevisiae* null mutants, including *osh2*Δ, *osh3*Δ, *osh6*Δ, *lam1*Δ, *ysp2*Δ, *sip3*Δ, *lam4*Δ, *lam5*Δ, and *lam6*Δ, to OTB. Loss of the *OSH2*, *OSH3*, *OSH6*, *YSP2*, *LAM4*, *LAM5*, and *LAM6* genes increased the susceptibility of *S. cerevisiae* to OTB. By contrast, deleting the *LAM1* and *SIP3* genes reduced the susceptibility of *S. cerevisiae* to OTB (Figure 5b). To further verify the sterol transport mechanism in fungal pathogens, we constructed sterol transporter gene‐deleted strains (*OSH2*, *OSH3*, *OSH4*, *OSH7*, *YSP2*, *SIP3*) in *C. albicans* SN152. Compared to the wild‐type strain, the *sip3*Δ/Δ null mutants exhibited resistance to OTB determined by spot assay (Figure 5c). Taken together, these findings suggest that the inhibitory effect of OTB on the PM localization of ergosterol depends on Sip3.

### 2.6 OTB Targeted Sec31 and Disrupted the Function of Sip3

**Figure 6:**
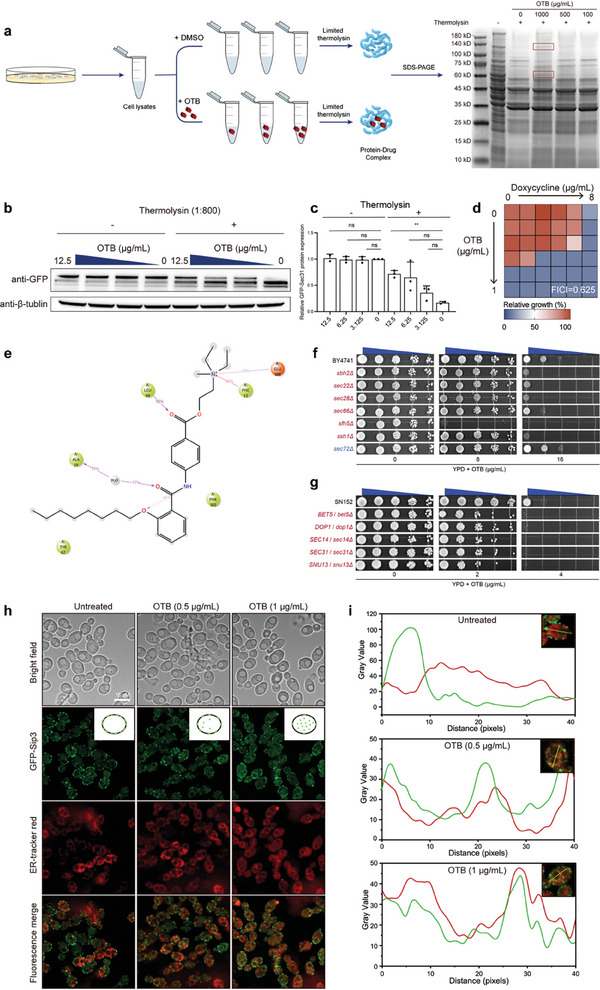
OTB disrupts the normal localization of the Sip3 protein by binding to the Sec31 protein. a) Schematic overview of DARTS and DARTS results from *C. albicans* protein extracts treated with OTB. *C. albicans* (SC5314) lysates were incubated with indicated drugs, subjected to thermolysin digestion, and Coomassie (SimplyBlue)‐staining. Red boxes indicate OTB‐protected proteins from thermolysin proteolysis. b) Western blot results using anti‐GFP and anti‐β‐tubulin antibodies. The DARTS assay shows that OTB protects Sec31, but not β‐tubulin, from degradation. c) Quantification of the signal intensity ratio between different concentrations of OTB using antibodies against GFP and β‐tubulin. Error bars represent the standard errors of three independent experiments. Significance determined by one‐way ANOVA with Bonferroni's multiple comparisons test; *p*‐value: **<0.01. *p*‐value, compared to untreated: 0.0018. d) Dose‐matrix titration assays with doxycycline in combination with OTB in P*_ADH1_*‐*SEC31*::*SEC31*/*sec31*∆ mutant strains were performed as previously described. e) Schematic detailing ligand atom interactions with protein residues. f) Spot assays of OTB were performed against wild‐type BY4741 and null mutant strains of *SBH2*, *SEC22*, *SEC28*, *SEC66*, *SFH5*, *SSH1*, and *SEC72*. g) Spot assays of OTB were performed against wild‐type SN152 and mutant strains of *BET5*/*bet5*, *DOP1*/*dop1*, *SEC14*/*sec14*, *SEC31*/*sec31*, and *SNU13*/*snu13*. h) Fluorescence localization of the Sip3 protein at the ER, with corresponding bright field images, ER tracker staining images, and merged images. Assays were performed in biological triplicate. Scale bar, 10 µm. i) The intensity distribution curves of GFP green fluorescence and ER tracker red fluorescence of the representation of single cells.

The present study suggests that OTB may selectively target ergosterol transporters, such as Sip3, or proteins involved in the regulation of these transporters, leading to the disruption of ergosterol localization in the PM. A drug affinity responsive target stability (DARTS) analysis, which is widely used for identifying and investigating interactions between proteins and ligands, is based on the fact that the binding of a small molecule to a protein can induce structural stabilization, thereby conferring on the target protein resistance to proteases.^[^ ^35^ ^]^ In this study, we employed DARTS analysis to investigate the specific proteins interacting with OTB. DARTS analysis revealed distinct protected bands at ≈130 kDa and 60 kDa in the proteolyzed extracts of OTB‐treated cells **(red rectangle indicated bands in lane 4 in** **Figure** **6**a). Given the potential for proteins in the lower molecular weight range to result from the degradation of proteins in the higher molecular weight range, our initial approach used liquid chromatography‐mass spectrometry (LC‐MS) for the analysis of the higher molecular weight band. A comprehensive analysis revealed the presence of 59 proteins falling within the designated size range (120‐140 kDa) in the higher molecular weight band **(Table** **S1**). Among this set of proteins, we identified Sec31 as a prime candidate target for OTB, given its close association with the PM localization of ergosterol. Sec31, a constituent of the coat protein complex II(COPII), plays a crucial role in the formation of a protective coat on the surface of vesicles emerging from the ER. This coat serves to facilitate the efficient packaging and sorting of proteins and lipids into vesicles, thereby enabling their transport.^[^ ^36^ ^]^

To further verify the binding of OTB to Sec31, we generated a mutant in which the N‐terminal of Sec31 was tagged with a green fluorescence protein (GFP). The results obtained from DARTS assays unequivocally demonstrated that OTB effectively shielded Sec31 from degradation induced by thermolysin (Figure 6b,c). Furthermore, a P_tet‐off_‐*SEC31*/*sec31*Δ mutant was generated, allowing suppression of the *SEC31* gene in the presence of doxycycline (DOX). An additive relationship between OTB and DOX was observed when tested against the P_tet‐off_‐*SEC31*/*sec31*Δ mutant (FICI = 0.625), providing additional evidence that Sec31 serves as the target protein of OTB (Figure 6d).

To provide additional clarification on the interaction between OTB and Sec31, we employed virtual docking and molecular dynamics simulations. The outcome of the virtual docking analysis revealed the direct formation of multiple sets of interacting forces between Sec31 and OTB. The calculated binding energy of the Sec31‐OTB complex was −8.0 kcal mol^−1^, indicating a highly favorable interaction (Figure S3a, Supporting Information). In the analysis of molecular dynamics simulations, we observed that the initial conformations of the protein and small molecule exhibited a high level of stability (Figure S3b‐d, Supporting Information). Following the dynamic simulations, a more stable binding conformation was formed based on the existing framework (Figure S3b‐d, Supporting Information). Among the various sets of interacting forces established between the protein and small molecule, the frequency of hydrogen bond formation involving LEU69 reached a remarkable 95% (Figure 6e; Figure S3e,f, Supporting Information).

To further investigate whether OTB impairs vesicle‐mediated trafficking, we tested the susceptibility to OTB of *S. cerevisiae* gene deletion mutants, including *sbh2*Δ, *sec22*Δ, *sec28*Δ, *sec66*Δ, *sfh5*Δ, *ssh1*Δ, and *sec72*Δ, that are known to be involved in vesicle‐mediated trafficking. We found that the deletion of these genes led to an increased susceptibility of *S. cerevisiae* to OTB (Figure 6f). We further generated heterozygous deleted mutants for a variety of vesicle‐mediated trafficking genes in *C. albicans*, including *SEC14/sec14*Δ, *DOP1/dop1*Δ, *BET5/bet5*Δ, *SUN13/sun13*Δ, and *SEC31/sec31*Δ, all of which exhibited heightened sensitivity compared to the wild‐type *C. albicans* (Figure 6g). These findings support the model that OTB had the capacity to hinder vesicle‐mediated trafficking.

Given that Sip3 is a membrane protein, OTB may hinder vesicle‐mediated trafficking by inhibiting Sec31. We propose that OTB may inhibit the localization of Sip3 on the ER membrane, leading to the loss of ergosterol transport function and subsequent accumulation of ergosterol within *C. albicans* cells rather than at the PM. To test this idea, we generated a mutant strain wherein the N‐terminus of Sip3 was fused with a GFP tag. The ER was marked using ER‐tracker red as an ER probe. We used laser confocal analysis and found that OTB inhibited the localization of Sip3 to the ER membrane. Instead, OTB caused the diffusion of Sip3 into the ER cavity, consequently leading to the impairment of its ergosterol transport function (Figure 6h,i).

### 2.7 OTB Triggered Autophagy in *C. Albicans*

**Figure 7:**
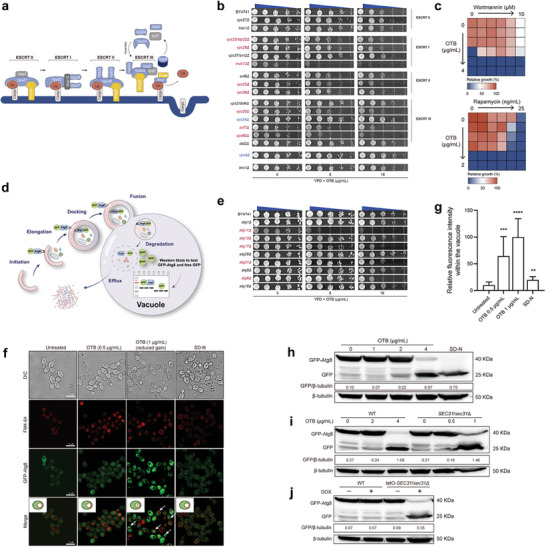
OTB triggers autophagy in *S. cerevisiae* and *C. albicans*. a) Schematic representation of the ESCRT complex composition. b) Spot assays of OTB were performed against wild‐type BY4741 and null mutant strains of *VPS27*, *HSE1*, *VPS23*, *VPS28*, *VPS37*, *MVB12*, *SNF8*, *VPS25*, *VPS36*, *VPS2*, *VPS20*, *VPS24*, *SNF7*, *VPS60*, DID2, *VPS4*, and *BRO1*. c) Dose‐matrix titration assays with wortmannin and rapamycin in combination with OTB separately. d) Schematic overview illustrating Atg8 degradation, a marker of autophagy, and its detection through immunoblot analysis. e) Spot assays of OTB were performed against wild‐type BY4741 and null mutant strains of *ATG1*, *ATG11*, *ATG13*, *ATG17*, *ATG29*, *ATG31*, *ATG8*, *ATG9*, and *ATG18*. f) Confocal micrographs of vacuole staining (FM4‐64) and localization of Atg8 proteins in the GFP‐Atg8 mutant cells following the indicated treatment. DIC is Differential Interference Contrast; FM4‐64 was visualized as red fluorescent; Atg 8 proteins were visualized as green fluorescent. Assays were performed in biological triplicate. Scale bar, 10 µm. g) Quantification of GFP intensity within the vacuole is shown in (f). Data are expressed as mean ± SD for biological triplicates. Significance determined by one‐way ANOVA with Bonferroni's multiple comparisons test; *p*‐value: **<0.01, ***<0.001, ****<0.0001. h) Immunoblot analysis of GFP‐Atg8 proteolysis in GFP‐Atg8 cells following the indicated treatment. Degradation rates were calculated using the formula: GFP:β‐tubulin. i) Immunoblot analysis of GFP‐Atg8 proteolysis in GFP‐Atg8 wild type and GFP‐*ATG8*:: *SEC31*/*sec31*∆ cells following the indicated treatment. Degradation rates were calculated using the formula: GFP:β‐tubulin. j) Immunoblot analysis of GFP‐Atg8 proteolysis in GFP‐Atg8 wild type and GFP‐*ATG8*:: *SEC31*/*sec31*∆:: P*_ADH1_*‐*SEC31* cells treated with doxycycline or without as indicated. Degradation rates were calculated using the formula: GFP:β‐tubulin.

Because vesicle‐mediated trafficking appears impaired by OTB, and the endosomal sorting complex required for transport (ESCRT) complex plays an essential role within the endosomal system, we tested the effects of deletions of ESCRT‐related genes on the susceptibility of *S. cerevisiae* to OTB. The absence of the *VPS23* and *VPS28* genes, constituents of ESCRT complex I, the *VPS25* and *VPS36* genes, constituents of ESCRT complex II, and the *VPS20*, *SFN7*, and *VPS60* genes, constituents of ESCRT complex III, all heightened the susceptibility of *S. cerevisiae* to OTB (**Figure** **7**a,b). These results suggest a potential association between the antifungal mechanism of OTB and the vesicular transport process mediated by the ESCRT complex.

The ESCRT complex participates in the sorting and transport of proteins into vesicles that form multivesicular bodies (MVBs) and trigger autophagy. Therefore, we speculated that OTB may trigger autophagy in *C. albicans*. In support of this hypothesis, OTB enhances the antifungal efficacy of rapamycin, an established autophagy inducer, while counteracting the antifungal effects of wortmannin, a known autophagy inhibitor (Figure 7c). Over 20 of these autophagy‐related (ATG) proteins are indispensable for autophagosome formation and are categorized into several functional groups: the Atg1 kinase complex, the transmembrane protein Atg9, an autophagy‐specific phosphatidylinositol 3‐kinase (PI3K) complex, the Atg2‐Atg18 complex, and the Atg8 and Atg12 conjugation systems.^[^ ^37^ ^]^ The Atg1 kinase complex, positioned as the most upstream component, serves as a key initiator of autophagy. In response to starvation, Atg1 is recruited to phagophore assembly site (PAS) sites, triggering autophagy.^[^ ^37b^ ^]^ This complex comprises 6 proteins in *S. cerevisiae*: Atg1, Atg11, Atg13, Atg17, Atg29, and Atg31. The assembly of the Atg1 complex enhances the kinase activity of Atg1, which is crucial for the regulation of autophagy (Figure 7d).^[^ ^38^ ^]^ Loss of four (Atg11, Atg13, Atg17, and Atg 31) out of these six proteins increased the susceptibility of *S. cerevisiae* to OTB (Figure 7e).

To further establish that OTB triggers autophagy in *C. albicans*, we generated a mutant where the N‐terminal of Atg8 was tagged with GFP. Atg8 is crucial for the autophagic machinery, playing a key role in inducing autophagy and forming PAS autophagosomes. Upon autophagy activation, GFP‐Atg8 translocates along autophagosomes and is released into vacuoles. GFP exhibits strong resistance to vacuolar hydrolases, which leads to Atg8 degradation and GFP retention, providing the conversion of GFP‐Atg8 to GFP as a measure of autophagic activity (Figure 7d).^[^ ^37^ ^]^ The wild‐type strain displayed a ubiquitous distribution of GFP‐Atg8 throughout the cells, with nearly 100% of cells accumulating this fusion protein. In contrast, following OTB treatment or transfer to nitrogen‐starvation conditions, GFP‐Atg8 translocated into vacuoles (Figure 7f,g). Immunoblotting of GFP‐Atg8 and GFP was also conducted to confirm these results. Consistent with the GFP fluorescence observations, immunoblotting reveals that OTB increased the levels of GFP fragments (Figure 7h).

The induction of autophagy has been found to result in the removal of mitochondria and the impairment of mitochondrial functions in various organisms.^[^ ^39^ ^]^ Since OTB has been shown to induce autophagy in *C. albicans*, it was hypothesized that OTB would also negatively affect mitochondrial function in this organism. One characteristic of mitochondrial dysfunction in *C. albicans* is the increased production of reactive oxygen species (ROS), which was indeed observed upon OTB treatment (Figure S4a, Supporting Information). Furthermore, the detrimental impact of OTB on mitochondrial function in *C. albicans* is evident in its enhanced antifungal activity in the non‐fermentative carbon source (glycerol) medium compared to the fermentative carbon source (glucose) medium (Figure S4b,c, Supporting Information).

Given that Sec31 is the target protein of OTB, a lower concentration of OTB (1 µg mL^−1^) elicited the degradation of GFP‐Atg8 in the *SEC31*/*sec31*∆ mutant strains (Figure 7i). Similarly, the inhibition of Sec31 expression should result in a phenotype like that observed in *C. albicans* treated with OTB, inducing autophagy. Indeed, the degradation of GFP‐Atg8 is induced by DOX through the inhibition of *SEC31* gene expression in the P_tet‐off_‐*SEC31*/*sec31*Δ mutant (Figure 7j).

### 2.8 *C. Albicans* Exhibited a Low Propensity for Developing Resistance to OTB

**Figure 8:**
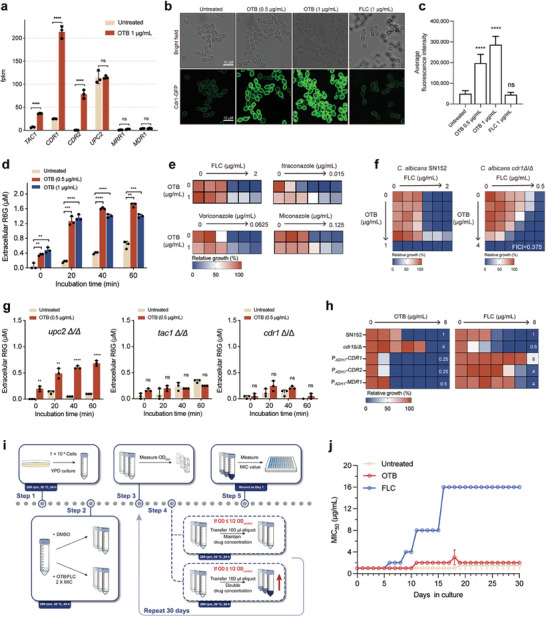
OTB activates Cdr1 but is less likely to induce resistance in *C. albicans*. a) Analysis of the expression of *TAC1*, *CDR1*, *CDR2*, *UPC2*, *NDT80*, *EFG1*, *MRR1*, and *MDR1* in the RNA‐seq results. Significance was determined by one‐way ANOVA with Bonferroni's multiple comparisons test; *p*‐value: ****<0.0001. b) Confocal micrographs of membrane localization of Cdr1‐GFP in the Cdr1‐GFP mutant following the indicated treatment. Assays were performed in biological triplicate. Scale bar, 10 µm. c) Quantification of GFP intensity is shown in (b). Data are expressed as mean ± SD for biological triplicates. Significance determined by one‐way ANOVA with Bonferroni's multiple comparisons test; *p*‐value: ****<0.0001. d) Extracellular rhodamine 6G (R6G) concentrations in the presence of OTB (0.5 or 1 µg mL^−1^) against *C. albicans* (SN152). Values are the mean ± standard deviation (indicated by error bars) from three independent experiments. Significance determined by one‐way ANOVA with Bonferroni's multiple comparisons test; *p*‐value: **<0.01, ***<0.001, ****<0.0001. e) Dose‐matrix titration assays with fluconazole, voriconazole, itraconazole, and miconazole in combination with OTB. f) Dose‐matrix titration assays with fluconazole in combination with OTB against the null mutant strain of *CDR1*. g) Extracellular rhodamine 6G (R6G) concentrations in the presence of OTB (0.5 or 1 µg mL^−1^) against the null mutant strain of *UPC2*, *TAC1*, and *CDR1* separately. Values are the mean ± standard deviation (indicated by error bars) from three independent experiments. Significance determined by one‐way ANOVA with Bonferroni's multiple comparisons test; *p*‐value: **<0.01, ****<0.0001. h) Dose‐matrix titration assays with OTB and fluconazole separately against (1) wild‐type SN152; (2) the null mutant strains of *CDR1*; (3) the overexpression strains of *CDR1*, *CDR2*, and *MDR1*. i) Schematic overview of the in vitro experimental evolution assay. j) Variations in OTB and FLC MICs for *C. albicans* (SN152) grown in YPD medium containing different concentrations of drugs. Values are the mean ± standard deviation (indicated by error bars) from three independent experiments.

Azoles exert their inhibitory effect on ergosterol synthesis, reducing intracellular ergosterol levels. This reduction in intracellular ergosterol content has been observed to trigger the activation of Upc2, subsequently activating the transcription regulatory factor Tac1 and its associated efflux pump proteins.^[^ ^40^ ^]^ Because OTB hinders the membrane localization of ergosterol and induces a state of relative ergosterol deficiency, we propose that OTB may also activate the efflux proteins of *C. albicans*. The RNA‐seq results obtained in this study demonstrate that OTB induced a significant upregulation in the expression levels of the *CDR1* and *CDR2* genes, as well as the *TAC1* gene, which encodes the translation regulator Tac1 responsible for regulating the expression of the *CDR1* and *CDR2* genes (**Figure** **8**a). To further investigate the impact of OTB on Cdr1, we employed a GFP tag to label the C‐termini of Cdr1 and utilized laser confocal microscopy to observe changes in Cdr1 abundance.^[^ ^41^ ^]^ Our findings reveal that, in the presence of OTB (1 µg mL^−1^), the amount of Cdr1 notably increased, particularly at the PM, when compared to the control condition with FLC (1 µg mL^−1^) (Figure 8b,c). Furthermore, a rhodamine 6G efflux assay was employed to assess the impact of OTB on the efflux activity of Cdr1. Notably, the content of rhodamine 6G in extracellular was significantly increased (indicating enhanced efflux) when treated with varying concentrations of OTB compared to the untreated control group (Figure 8d). In the dose‐matrix titration assays conducted on OTB and azoles, including FLC, miconazole, itraconazole, and voriconazole, it was observed that the activation of OTB on the function of Cdr1 efflux protein resulted in an antagonistic effect on the antifungal activity of azoles at a sub‐inhibitory concentration of 0.5 µg mL^−1^ (Figure 8e). However, OTB and FLC showed synergistic antifungal activity against the *cdr1*Δ/Δ null mutant (FICI = 0.375) (Figure 8f). These findings provide evidence that OTB effectively activated the efflux function of Cdr1.

Our findings demonstrate that deleting the *TAC1* and *CDR1* genes effectively impedes the promotion of R6G efflux by OTB. However, the absence of the *UPC2* gene did not have the same inhibitory effect (Figure 8g). This outcome suggests that OTB did not activate Upc2 by reducing cellular ergosterol levels and activating Cdr1 as azoles do. Conversely, OTB hindered the transportation of ergosterol to the PM, increasing intracellular ergosterol concentration. The subsequent activation of Cdr1, induced by the excessive accumulation of ergosterol, serves to mitigate the deleterious consequences resulting from elevated ergosterol levels.^[^ ^42^ ^]^

Although azoles can inhibit the synthesis of ergosterol, azoles can be transported by Cdr1 outside the cell, allowing *C. albicans* to tolerate azoles and ultimately develop resistance. Indeed, loss of the *CDR1* gene increased the susceptibility of *C. albicans* to FLC, and ectopic over‐expression of the *CDR1*, *CDR2*, and *MDR1* genes decreased the susceptibility of *C. albicans* to FLC (Figure 8h). Interestingly, the *cdr1*Δ/Δ null mutant decreased the susceptibility to OTB (MIC values increased from 1 to 4 µg mL^−1^), while the P*_ADH1_*‐*CDR1*, P*_ADH1_*‐*CDR2*, and P*_ADH1_*‐*MDR1* ectopic over‐expressed mutants caused increased susceptibility (MIC values decreased from 1 to 0.25 µg mL^−1^ for *CDR1* and *CDR2* gene, and from 1 to 0.5 µg mL^−1^ for *MDR1* gene) (Figure 8i). These findings indicate that OTB did not serve as a substrate for the efflux pump Cdr1, which suggests that OTB can be employed to combat *C. albicans* isolates that are resistant to azole drugs.

Given that Cdr1 does not recognize OTB as a substrate, we wondered if *C. albicans* may have difficulty acquiring resistance to OTB. To investigate this, *C. albicans* cells were subjected to OTB or FLC at concentrations twice their MIC values. The MIC value of FLC against *C. albicans* increased from 1 to 2 µg mL^−1^ after six passages, corresponding to six days of FLC exposure. Subsequently, the highest MIC value of FLC against *C. albicans* reached 16 µg mL^−1^ after 16 days of FLC exposure and remained consistent from day 16 to day 30. In contrast, the MIC value of OTB against *C. albicans* exhibited an increase from 1 to 2 µg mL^−1^ after 11 days of OTB exposure. Furthermore, the MIC values of OTB reached 4 µg mL^−1^; this required 18 days of uninterrupted OTB exposure and reverted to 2 µg mL^−1^ on the 19th day of exposure (Figure 8j).

## 3 Discussion and Conclusion

**Figure 9:**
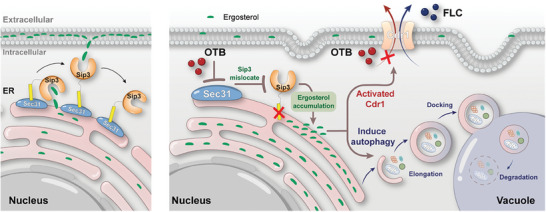
Schematic overview of the mechanism illustrating the antifungal activity of OTB.

In this study, we find that OTB has broad‐spectrum and potent antifungal activity and can inhibit hyphal and biofilm formation while demonstrating minimal toxicity, and thus appeared as a promising candidate for antifungal therapy. Further investigation revealed that OTB hindered vesicle‐mediated trafficking by targeting Sec31, leading to the inhibition of the PM localization of the ergosterol transporter Sip3. As a consequence, OTB disrupted the PM localization of ergosterol and induced cytotoxic autophagy. Because OTB did not appear to be exported by Cdr1, a classic pathway for drug resistance in *C. albicans* is not available for OTB (**Figure** **9**). We found that disrupting the distribution of ergosterol to the PM is a useful antifungal strategy. Azoles effectively combat candidiasis by targeting lanosterol 14‐α‐demethylase to inhibit ergosterol synthesis.^[^ ^2^ ^]^ However, lanosterol 14‐α‐demethylase belongs to the cytochrome P450 family and resembles hepatic drug‐metabolizing enzymes found in the human liver.^[^ ^43^ ^]^ Hepatotoxicity is a known adverse effect of azoles, rendering them poorly tolerated by numerous patients, particularly those with compromised liver function.^[^ ^44^ ^]^ Hindering the localization and integration of ergosterol into the PM, without inhibiting the enzymes associated with ergosterol synthesis, may effectively impede the growth and proliferation of pathogenic fungi while avoiding hepatotoxicity. A recent study has provided evidence that the absence of Ysp2, a retrograde ergosterol transporter, disrupts the distribution of ergosterol and reduces the virulence of *C. neoformans*.^[^ ^4a^ ^]^ The present study demonstrated the inhibitory impact of OTB on the appropriate PM localization of ergosterol, leading to the potent antifungal activity of OTB. We further found that OTB hindered the PM localization of ergosterol by deactivating the functionality of ergosterol transporters, such as Sip3. OTB dysfunctions ergosterol by hindering its localization on the PM, thereby evading the adverse consequences associated with direct inhibition of enzymes implicated in ergosterol synthesis as azoles do.

Inducing cytotoxic autophagy in fungal cells is a novel and practical antifungal strategy. Autophagosomes, originating from sites associated with the ER, play a vital role in the cellular recycling process. Due to the scarcity of transmembrane proteins on the autophagosome membrane, it is believed that lipid transfer, lipid modifications, and membrane‐associated proteins primarily regulate the formation of autophagosomes.^[^ ^45^ ^]^ In mammalian cells, the initiation of autophagy and the enhancement of autophagy flux during periods of starvation are facilitated by the reduction of cholesterol levels and the cholesterol transport protein GRAMD1C.^[^ ^45^, ^46^ ^]^ Our findings indicate that OTB targeted Sec31 and disrupted vesicle‐mediated trafficking. Consequently, the impairment of vesicle‐mediated trafficking leads to complications in the membrane localization of membrane proteins, especially the ergosterol transporter Sip3, ultimately resulting in their accumulation within the ER. Sip3 dysfunction resulted in the intracellular buildup of ergosterol metabolic intermediates, thereby inducing cytotoxic autophagy. In the present study, we developed a novel antifungal concept whereby the depletion of ergosterol in the PM induces cytotoxic autophagy.

*C. albicans* face difficulties in developing resistance to OTB. The primary mechanism employed by *C. albicans* to acquire azole resistance involves the activation of efflux pump proteins, notably Cdr1, which facilitates the export of drugs from the cell, thereby reducing intracellular drug levels.^[^ ^5^ ^]^ Fortunately, it has been determined that OTB did not serve as a substrate for Cdr1, thereby preventing the activation of Cdr1 from reducing the antifungal efficacy of OTB. Our investigation has revealed that the overexpression of Cdr1 can actually augment the potency of OTB against *C. albicans*, as it led to further depletion of ergosterol and its intermediates.^[^ ^42^ ^]^ The results of this study indicated that the utilization of efflux pump proteins by *C. albicans* as a defense mechanism against the antifungal properties of drugs was ineffective when confronted with OTB. The characteristics of OTB provided valuable information regarding the desirable attributes of potential antifungal medications, including a reduced affinity for Cdr1 and a failure to be removed from the cell by Cdr1, thus hindering the development of antifungal drug resistance in *C. albicans*.

We identified Sec31 as the target protein for OTB in the present study. OTB is classified as a quaternary ammonium salt compound based on its structural composition. Numerous studies have provided evidence that the positively charged head group in quaternary amine compounds can undergo electrostatic attraction with the negatively charged surface of microorganisms. This interaction forms an electrostatic bond, augmenting the pressure exerted on the cell wall. Consequently, the penetration of long‐chain alkyl into the PM is facilitated, ultimately leading to the leakage of intracellular contents and subsequent cell death.^[^ ^23^, ^47^ ^]^ However, this study has determined that OTB did not cause direct harm to the cell membrane, distinguishing it from other quaternary ammonium salt compounds that exert antimicrobial effects through PM damage. Additionally, investigating compounds containing quaternary ammonium salt groups revealed that only OTB exhibited noteworthy antifungal activity (Figure S5, Supporting Information). The antifungal activity of OTB analogs lacking quaternary ammonium salt groups was subjected to further investigation. We found that none of these compounds exhibited antifungal activity and had MIC values exceeding 64 µg mL^−1^ (Figure S6, Supporting Information). Based on DARTS and chemogenomic analysis, it appears that Sec31 serves as the primary target of OTB. Furthermore, molecular dynamic simulation experiments provided insights into the importance of carbonyl and quaternary ammonium salt groups in facilitating the interaction between OTB and Sec31.

In conclusion, this study presents a novel antifungal strategy through the elucidation of the antifungal mechanism of OTB. This mechanism involves impeding the transportation of ergosterol to the PM and inducing cytotoxic autophagy. The utilization of OTB as a representative of this new strategy offers the advantage of circumventing hepatic toxicity associated with azole‐induced liver enzyme inhibition while also preventing the emergence of drug resistance in *C. albicans* by excluding drug pumps. These novel findings pave the way for further advancements in the field of antifungal therapy.

## 4 Experimental Section

### Strains, Primers, Agents, and Culture Conditions

All strains, primers, and compounds used in this study are listed in Tables S2, S3, and S4, respectively. All strains were cultured in YPD (1% [W/V] yeast extract [Oxoid Ltd, LP0021B], 2% [W/V] peptone [Oxoid Ltd, LP0137], and 2% [W/V] glucose [Sangon Biotech, A6120219]) for liquid culture growth or YPD with 2% (W/V) agar for solid medium growth at 30 °C unless otherwise specified. To construct mutant strains, we used a synthetic complete dropout medium (0.67% [W/V) yeast nitrogen base [Sangon Biotech, A610507] without amino acids, 2% [W/V] glucose, 2% [W/V] agar [Sangon Biotech, A505255], and appropriate amino acid mix) to screen positive colonies. SDA (1% [W/V] peptone and 4% [W/V] glucose) medium supplemented with 2% (W/V) agar was used as culture media for all plate counting assays. In the hyphae growth assay and XTT reduction assay, RPMI 1640 medium (buffered to a pH of 7.0 with 0.165 M MOPS; Sigma‐Aldrich, R4130) was used as culture media. A yeast deletion library encompassed 4848 single knockouts of non‐essential genes replaced with the *KanMX* cassette (Open Biosystems, YSC1053). Mutants from the yeast deletion library were routinely grown on the YPD medium with 200 µg mL^−1^ of G418 sulfate (Agri‐Bio System, 3000) for deletant strains, supplemented with 2% (W/V) agar when solid media were required for plating.

### High‐Throughput Chemical Screen

A collection of 2372 compounds from the FDA‐approved drugs library (Med Chem Express, HY‐L022) was used to identify compounds that exhibit inhibition of fungal growth and may possess novel mechanisms of action. These compounds were stored as 10 mM stocks in DMSO (Sangon Biotech, A503039) and subjected to screening at a final concentration of 10 µM. In each well of a 96‐well plate, a 200 µL suspension of *C. albicans* cells (1×10^3^ cells mL^−1^) and a compound at a concentration of 10 µM were added. Subsequently, the plates were incubated at 30 °C for 24 h. The growth of *C. albicans* was then assessed by measuring the absorbance at 600 nm using a spectrophotometer (Thermo Fisher Scientific, Multiskan SkyHigh, USA). A compound is deemed a potential candidate for further investigation if the optical density (OD) value of the well containing the compound is less than 10% of the OD value of the well without a drug.

### Dose‐Matrix Titration Assays

Dose‐matrix titration assays were used to evaluate drug synergies as described previously.^[^ ^48^ ^]^ Briefly, 50 µL of fourfold the final drug concentration of drug A was dispensed in two‐fold serial dilution steps across plate columns. Then, 50 µL of fourfold the final drug concentration of drug B was dispensed in two‐fold serial dilution steps across rows of the plate. 100 µL of overnight *C. albicans* cultures adjusted to 1×10^3^ cells mL^−1^ was dispensed in all drug‐containing wells plus one control well containing no drugs. Subsequently, these plates were incubated at 30 °C, and growth was measured by absorbance at 600 nm after 24 h. All assays were performed and repeated three times.

### Spotting Assay

The spotting assay was conducted using the method previously described.^[^ ^28^ ^]^ In summary, mid‐log‐phase cultures of fungal strains relevant to the experimental conditions were grown overnight and subsequently adjusted to a concentration of 1 × 10^7^ cells mL^−1^ using a hemacytometer. These cultures were then serially diluted 1:10 in sterile PBS and spotted onto designated plates containing specified concentrations of compounds. Following a 48‐hour incubation period at 30 °C, photographs were taken capturing the cellular growth.

### Growth Inhibition Curve Assays

The growth rate was determined using the methods previously described.^[^ ^49^ ^]^ In brief, *C. albicans* cells were cultured in a YPD medium until reaching the exponential growth phase. The *C. albicans* cell suspension was then diluted to an OD_600_ of 0.1 in 150 µL of YPD medium, either with or without various concentrations of OTB, in 96‐well plates. The plates were incubated at 30 °C with continuous shaking using a TECAN plate reader (TECAN, Infinite F200 PRO, Switzerland). The OD_600_ was measured every 15 min for 48 h using TECAN i‐control software. Growth curves were generated for three biological replicates. Data are plotted in Prism v9.3.0 (GraphPad).

### Time‐Kill Curve Assay

The *C. albicans* cells were cultured in a YPD medium until they reached the exponential growth phase. *C. albicans* suspension was diluted with a YPD medium to an OD_600_ of 0.2 in 10 mL, with or without varying concentrations of OTB. For the time‐kill curve assay of AmB (Shanghai Aladdin Biochemical Technology, A657418) following OTB treatment, the tubes with OTB were incubated at 30 °C in a shaker at 200 rpm for 4 h. Subsequently, the fungal concentration in all groups was adjusted to an OD_600_ of 0.2, and 1 µg mL^−1^ Am B was added. The tube was then incubated at 30 °C in a shaker set at 200 rpm. At 0, 2, 4, 8, 12, 24, and 48 h of incubation, a sample of the mixture was taken and diluted with sterile PBS. The diluted sample was then plated on an SDA solid medium to determine CFUs. Each sample group was replicated three times.

### Hypha Growth Assay

*C. albicans* cells were cultured in a YPD medium until they reached the exponential growth phase. Subsequently, they were collected, washed three times with PBS, and then diluted with RPMI 1640 medium to 1× 10^6^ cells mL^−1^. A volume of 100 µL of this suspension was added to each well of a 96‐well plate. Different concentrations of OTB were added to the wells, with FLC (Shanghai Aladdin Biochemical Technology, E129360) serving as a control. The plates were then incubated at 37 °C for 4 h. Morphological differences between the groups treated with drugs and those without drug treatment were observed and recorded using an inverted microscope (Motic, AE2000, China).^[^ ^49^ ^]^

### XTT Reduction Assays

A semiquantitative measure of biofilm formation was calculated by using an XTT ((sodium 3′‐[1‐(phenylamino‐carbonyl)−3,4‐tetrazolium]‐bis [4‐methoxy‐6‐nitro] benzene sulfonic acid hydrate); Macklin reagent, X820547}‐reduction assay, adapted from previous reports.^[^ ^49^ ^]^ The assay is based on the cleavage of the yellow tetrazolium salt XTT to form an orange formazan dye by metabolically active cells. *C. albicans* cultures were resuspended in RPMI 1640 medium buffered with MOPS (Sangon biotech, A100670) to a final concentration of 10^6^ cells mL^−1^. An aliquot of 100 µ L was added to each well of a 96‐well plate and incubated at 37 °C. After 90 min, wells were gently washed twice with 1× PBS to remove non‐adherent cells. The fresh RPMI 1640 medium was added with or without OTB or FLC. The RPMI 1640 medium was added to adhered cells without any drug for mature biofilm eradication assays and incubated for 24 h to establish mature biofilms. After the treatment of OTB or FLC for 24 h, non‐adherent cells were washed away with PBS, and biofilm cell metabolic activity was measured using the XTT reduction assay. Immediately before each assay, a phenazine methosulfate solution was prepared at a final concentration of 320 µg mL^−1^ and filter‐sterilized. Briefly, 90 µ L of XTT at 0.5 mg mL^−1^, and 10 µL of phenazine methosulfate (Shanghai Aladdin Biochemical Technology, P105579) at 320 µg mL^−1^ were added to each well, followed by incubation at 37 °C for 2 h in the dark. The absorbance of the supernatant was measured using a microplate reader (Thermo Fisher, Multiskan SkyHigh) at 450 nm. Experiments were conducted in at least three technical replicates for each strain/condition. Data are plotted in Prism v9.3.0 (GraphPad), showing the mean relative quantity ±SD.

### Confocal Laser Scanning Microscopy

To detect the upregulation of Cdr1 expression by OTB, overnight *C. albicans* Cdr1‐GFP cultures were diluted to an OD_600_ of 0.2 and grown in the presence of the respective compounds (or DMSO equivalent) for 4 h at 30 °C. Cells were then pelleted by centrifugation at 2500 g for 3 min and washed three times in PBS. GFP was visualized using confocal laser scanning microscopy (Leica Stellarissted, Germany) using a GFP Em535/Ex488 filter set and a YFP Em561/Ex532 filter set.^[^ ^41b^ ^]^

For filipin staining experiments, overnight *C. albicans* cultures were diluted to an OD_600_ of 0.2 and grown in the presence of respective compounds (or DMSO equivalent) for 4 h at 30 °C. Cells were then pelleted by centrifugation at 2500 g for 3 min and washed three times in PBS. All cells were covered with 15 µM filipin complex solution (Med Chem Express, HY‐N6716) and incubated at room temperature for 15 min. Before imaging, the cells were washed three times with PBS and then viewed under a UV filter set (Leica 63 × oil). The image was analyzed using Image J software.

To examine the localization of GFP‐Atg8, overnight GFP‐tagged cell cultures were diluted to an OD_600_ of 0.2 and grown in the presence of the respective compounds (or DMSO equivalent) for 4 h at 30 °C. Cells were then pelleted by centrifugation at 2500 g for 3 min and washed three times in PBS.1 mL of the suspension was mixed with 2 µL FM4‐64 (500 µg mL^−1^, prepared in DMSO; Med Chem Express, HY‐103466) and incubated at room temperature with gentle shaking for 30 min. The cells were then centrifuged, re‐suspended in 500 µL YPD, and incubated at 30 °C for a further 30 min. GFP was visualized using confocal laser scanning microscopy (Leica Stellarissted, Germany) using a GFP Em535/Ex488 filter set. All assays were done in biological triplicates shown in the figure, and the AOD of the fluorescence in the photo was analyzed using Image J.

### RNA Extractions and RNA Seq Analysis

*C. albicans* overnight cultures were diluted to an OD_600_ of 0.1 in 100 mL of fresh YPD medium and treated with 1 µg mL^−1^ of OTB or an equal volume of DMSO for 4 h. The Yeast RNA Extraction Kit (Zymo Research, R1002) was utilized to extract total RNA. The quality and integrity of the RNA were assessed using an Agilent 2100 bioanalyzer. Poly(a)‐containing mRNA was isolated from the total RNA with poly(T) oligonucleotide‐attached magnetic beads. The RNA obtained was fragmented and subjected to reverse transcription using random N6 primers. Subsequently, a bridge primer was employed to cyclize the single‐stranded DNA, yielding a library of single‐stranded circular DNA. Following the sequencing process, the raw data underwent quality trimming using SOAPnuke software (BGI Genomics, China). This involved removing reads with low quality (defined as a base with a mass value below 15 constituting more than 20% of the total bases in the read), joint contamination, and a high content of N (greater than 5%), which represents an unknown base. The resulting reads were considered clean. The differential expression of genes was assessed using HISAT2 software (v2.0.4) to compare the OTB treatment group with the control76. DEGs were identified based on the criteria of log2 fold change > 1 and *p*‐value < 0.05.

### Rhodamine 6G Efflux

Efflux of rhodamine 6G was determined using a previously described protocol.^[^ ^50^ ^]^ *C. albicans* cultures were diluted to an OD_600_ of 0.2 and grown in the presence of respective compounds (or DMSO equivalent) for 4 h at 30 °C. Pelleted cells were washed twice with PBS, and a 2% cell suspension corresponding to 1 × 10^8^ cells mL^−1^ was re‐suspended in PBS without glucose. Cells were then de‐energized for 1 h in PBS (without glucose). De‐energized cells were pelleted, washed, and re‐suspended in PBS without glucose, to which rhodamine 6G (Shanghai Aladdin Biochemical Technology, R105624) was added at a final concentration of 10 µM and further incubated for 30 min at 30 °C. Equilibrated cells with rhodamine 6G were washed and re‐suspended in PBS, which contained no glucose. Samples with a volume of 1 mL were withdrawn at specified times and were centrifuged at 9000 g for 1 min. At the indicated time intervals, energy‐dependent efflux was measured following the addition of 2% glucose to the cells re‐suspended in glucose‐free PBS. Glucose‐free negative controls were included in all experiments.

### Transmission Electron Microscopy Analysis

An electron microscopy fixation solution (2.5% glutaraldehyde; Sangon biotech, A600875) was added to *C. albicans* cells, which were then subjected to fixation in the dark at room temperature for 30 min. The samples were subsequently transferred to 4 °C and left overnight for further fixation. The sample was then fixed at room temperature for 2 h using a 1% osmic acid solution (Ted Pella Inc, 18 456) prepared in 0.1 M PBS (pH 7.4), followed by dehydration through an acetone (Sinopharm chemical reagent, 10 000 418) gradient ranging from 25% to 100%. The study employed a protracted infiltration procedure involving multiple applications of Durcupan ACM epoxy resin (SPI, 90529‐77‐4), followed by embedding and resin hardening at a temperature of 60 °C for 48 h. Subsequently, ultra‐thin sections measuring 80 nm were treated with a 2% uranyl acetate and 2.6% lead citrate solution for contrast enhancement and examined using a transmission electron microscope (HT7800, HITACHI, Japan).

### DARTS

DARTS experiment was conducted using a previously described protocol.^[^ ^51^ ^]^ For the DARTS experiment using yeast cell lysates incubated in vitro with OTB, *C. albicans* SC5314 cells were suspended in yeast lysis buffer (PBS [pH 7.2 to 7.6]) containing 1.0% protease inhibitor cocktail. Cell pellets were broken with glass beads for 30 s, 4 times at 4 °C in the Bead Ruptor12 system (OMNI International, USA). All steps were performed on ice or at 4 °C to help prevent premature protein degradation. Lysates were incubated with OTB from 100 to 1000 µg mL^−1^ or H_2_O control for 1 h at room temperature. Samples were then divided into two aliquots that underwent proteolysis with 10 µg of thermolysin (Sigma‐Aldrich, P1512) or PBS control for 15 min at room temperature. To stop the proteolysis, the reaction tubes were shifted to 4 °C, and EDTA (pH 8.0, 50 mM final concentration) and SDS/PAGE loading buffer were added to each sample. Aliquots of each sample were subjected to electrophoresis on 4 to 20% SDS/PAGE, and Coomassie staining was used to identify bands that specifically bind to OTB. Bands from SDS/PAGE gels were cut out and prepared for mass spectrometry analysis with trypsin digestion. LC‐MS analyzed peptides on a Thermo LTQ‐Orbitrap mass spectrometer with an Eksigent LC pump. For quantitative comparison of protein and peptide abundances, MS spectra were analyzed by using the differential workflow of Rosetta Elucidator (Rosetta Inpharmatics). Annotation was performed using PeptideTeller and ProteinTeller.

For the affinity experiments of OTB and Sec31, total proteins of GFP‐Sec31 were extracted using the method above. The cell lysates were incubated at room temperature with OTB or H_2_O in concentrations ranging from 3.125 to 12.5 µg mL^−1^ for 1 hour. Subsequently, 0.5 µg thermolysin was added per 45 µL of lysate protein and allowed to dissolve for 15 min. Proteins were separated by 4 to 20% SDS‐PAGE and transferred to a polyvinylidene difluoride (PVDF) membrane. After blocking, an anti‐GFP antibody or anti‐tubulin antibody was used for probing GFP‐tagged proteins or tubulin, which were then detected using the secondary antibody goat anti‐mouse IgG conjugated with horseradish peroxidase (HRP) and the Pierce ECL system (Thermo Fisher Scientific, USA).

### Western Blot Analysis

Total proteins of *C. albicans* cells treated with different conditions were extracted using the Bead Ruptor12 system in a lysis buffer consisting of PBS (pH 7.2 to 7.6) containing 5 mM EDTA (pH 8.0; Sangon biotech, B540625), 1 mM phenylmethylsulfonyl fluoride (PMSF, Sangon biotech, A610425), and 1.0% protease inhibitor cocktail (TargetMol, USA). The proteins were then separated using 4 to 20% SDS‐PAGE (Beyotime biotechnology, D0185S) and transferred onto a PVDF membrane (Beyotime biotechnology, FFP28). Following blocking, GFP‐tagged proteins or tubulin were probed using an anti‐GFP antibody (Santa Cruz Biotechnology, sc‐9996) or an anti‐tubulin antibody (Abbkine Scientific, ABL1030), respectively. Detection was achieved by employing a goat anti‐mouse IgG secondary antibody conjugated with HRP (Santa Cruz Biotechnology, sc‐516102) in conjunction with the Pierce ECL system (Thermo Fisher Scientific, USA).

### In Vitro Experimental Evolution Assay

The in vitro experimental evolution assay was conducted as previously described.^[^ ^52^ ^]^ In brief, *C. albicans* overnight cultures were resuspended in 5 mL YPD medium with compounds at a concentration of 2×MIC or DMSO equivalent, resulting in a final concentration of 1×10^6^ cells mL^−1^. Each condition was replicated in triplicate, resulting in three evolving populations per condition. Following a 24‐hour incubation period at 30 °C in a shaking incubator, the growth of individual populations was assessed in relation to the average growth of three control groups (DMSO groups) through spectrophotometric quantification using an OD _600_ measurement. Subsequently, aliquots containing 1×10^6^ cells were transferred into fresh YPD medium with a drug concentration equivalent to that of the previous culture when the OD_600_ of the evolving population was less than half of the OD_600_ of the average control or doubled the previous culture's drug concentration when the OD_600_ of the evolving population exceeded half of the OD_600_ of the average control. At each interval, a 0.5 mL portion of the suspension was combined with 0.5 mL of 80% glycerol (Sangon biotech, A501745), and the resulting mixture was subjected to freezing at −80 °C for conducting antifungal susceptibility testing.

### Evaluating the Antifungal Activity of OTB in a Mouse Infection Model

A standard protocol with a few modifications was used to establish a model of *C. albicans* infection in the gastrointestinal tract. For animal infections, 7‐ to 8‐week‐old female C57BL/6J mice (SLAC ANIMAL, China) were housed together with free access to food (standard rodent chow, autoclaved; LabDiet, 5010) and water. After 7 days of acclimation in the animal facility, all mice were given an intraperitoneal injection of cyclophosphamide (Shanghai Aladdin Biochemical Technology, C126044) 100 mg kg^−1^ for two consecutive days to keep the mice in an immunosuppressive state to create a fungal infection model. At the same time, the drinking water of the mice was replaced with a sufficient amount of antibacterial drinking water (levofloxacin sodium chloride injection diluted with triple sterile water, with a concentration of 0.4 g L^−1^) and changed daily throughout the entire experimental process. On the third day of immunosuppression, mice were orally gavaged with 4 × 10^8^ cells (0.2 mL volume). Two hours after administration of *C. albicans* cells, the mice in each group were intraperitoneally injected with cyclophosphamide again and given 50 mg kg^−1^ OTB or 1% DMSO by intragastric administration. Afterward, cyclophosphamide was administered on the third and sixth days of the observation period for supplementary immunosuppression, and compounds were given by intragastric administration every day until the end of the observation period. Mice showing extreme lethargy were considered moribund and were euthanized. Tongji University Animal Care Committee approved all experimental procedures involving animals. Kaplan‐Meier analyses were used to indicate the survival probabilities, and log‐rank testing was used to evaluate the significance of survival curves.

### PM Isolation and Sterol Analysis

A standard protocol with a few modifications was used to isolate PMs from suspensions of *C. albicans* treated with different compounds.^[^ ^53^ ^]^ Briefly, *C. albicans* overnight cultures were resuspended in lysis medium (1.2 M sorbitol, 0.1 M EDTA, 1% β‐Mercaptoethanol, 5 mg mL^−1^ zymolase) and incubated at 37 °C for 1 h. Protoplasts were then washed with 1.2 M sorbitol, lysed with ice‐cold ddH2O shock, and disrupted by sonication (5 s cycles for 2 min at 4 °C) using an ultrasonic processor (Scientz, China). Cell lysate was centrifuged at 1000 g at 4 °C for 10 min to remove unbroken material, and the supernatant was ultracentrifuged at 10 000 g at 4 °C for 60 min. The sterol analysis was performed by gas chromatography‐mass spectrometry (GC‐MS) with cholesterol as an internal standard, as previously described.

### Statistical Analysis

All experiments were repeated at least three times. Data represent biological replicates and meet the assumptions of the statistical tests described for each Figure. Results are expressed as the mean ± SD. Differences between experimental groups were assessed for significance using a two‐tailed unpaired Student's *t*‐test with GraphPad Prism 5 software.

### For Studies with Animals

Tongji University Animal Care Committee approved all experimental procedures involving animals. All experiments conformed with the Helsinki Declaration of 1975, as revised in 2008 (5) concerning Human and Animal Rights, and followed out policy concerning Informed Consent as shown on Springer.com. All institutional and national guidelines for the care and use of laboratory animals were followed.

## Conflict of Interest

The authors declare no conflict of interest.

## Author Contributions

C.Z. conducted most of the experiments and performed data analysis. C.Z., H. L., M.W., and Y.J. wrote the manuscript draft and revised the manuscript. C.Z., L.W., and Y.F. constructed mutant strains. C.Z., S.H., and J.Y. evaluated the antifungal activities, analyzed the ergosterol content, and collected data. C.Z., H. L., M. W., and Y. J. discussed and analyzed the data. H.L. and Y.J. conceived the idea. H.L. and Y. J. directed the experiments.

## Supporting information

Supporting Information

## Data Availability

The data that support the findings of this study are openly available in Sequence Read Archive at https://www.ncbi.nlm.nih.gov/bioproject, reference number 1012118.
