# Supplementary material for: Otilonium Bromide Exhibits Potent Antifungal Effects by Blocking Ergosterol Plasma Membrane Localization and Triggering Cytotoxic Autophagy in Candida Albicans
Source: Adv Sci (Weinh). 2024 Jul 12;11(35):2406473. doi: 10.1002/advs.202406473 (PMC11425263; doi:10.1002/advs.202406473)
Supplement: Supplementary file 1 — Supporting Information [file ADVS-11-2406473-s001.docx]

**Supplementary materials**

**1. Supplementary materials and methods**

**1.1 MIC assay**

Antifungal susceptibility testing was performed according to the Clinical and Laboratory Standards Institute (M27-A3), with a few modifications. In brief, compounds were diluted in 96-well fplates at two times the final concentration with a YPD medium. Subsequently, 100 μL of this dilution was combined with 100 μL *C. albicans* cell suspension (1×10^3^ cells/mL). The 96-well plates were subsequently incubated at 30°C under static conditions for 24 h. The absorbance of each well at a wavelength of 600 nm was measured using a spectrophotometer. The minimum drug concentration for growth inhibition exceeding 50% compared to the control well without compound is the MIC value of the compound. Data are plotted in Prism v9.3.0 (GraphPad).

**1.2 MFC assay**

The *C. albicans* cells were cultured in a YPD medium until they reached the exponential growth phase. These cells were then washed three times with PBS (Sango biotech, B040100) and diluted with YPD medium to obtain *C. albicans* suspension with a concentration of 1×10^4^ cells/mL. Subsequently, 100 μL of the *C. albicans* cell suspension was mixed with 100 μL of OTB (Med Chem Express, HY-B0499A) solutions with varying concentrations in 96-well plates. The plates were then incubated at 30°C under static conditions for 24 h. Then 200 μL of the *C. albicans* cell suspension from wells with no fungal growth were plated on SDA solid medium and incubated at 30°C for 48 h. The MFC refers to the lowest concentration of the OTB solution that results in the absence of CFUs on the SDA solid medium.

**1.3 CCK-8 viability assay**

Caco-2 intestinal epithelial cells were incubated in DMEM (HyClone Laboratories, SH30285.FS) containing 10% FBS (HyClone Laboratories, SH30070.01). The cytotoxic effect of OTB on Caco-2 viability was assessed by the cell counting kit-8 (CCK-8) assay.^[1]^ Briefly, Caco-2 (1 × 10^4^ cells/well) was seeded in 96-well microtiter plates and cultured in DMEM medium supplemented with 10% FBS at 37°C for 3 h for adhesion. After incubation, the supernatant was removed, and different concentrations of OTB (4 to 64 μg/mL) dissolved in fresh media without FBS were added. The plates were incubated for 24 h at 37°C. The CCK-8 solution (Target Molecule Corp, TP1197) was added to each well (10 μL/well) and incubated at 37°C for 2 h. The absorbance at 450 nm was measured using an ELISA plate reader. Cells incubated in DMEM with DMSO only (<1% of the total test volume) were set as the standard for 100% viability. The cytotoxic effect of OTB was determined by comparing the results to the control DMSO. The IC_50_ value represents the concentration that causes a 50% reduction in the initial cell amounts and is calculated by a nonlinear regression curve ﬁtting of triplicate determinations from dose-response assays against human cells (Caco-2). All experiments were performed in triplicate, and the positive control was FLC.

**1.4 Disruption of target genes**

Strains were constructed according to a standard protocol^[2]^that uses two-step fusion PCR to generate disruption fragments with relatively long (350 nucleotides) regions of homology with the target locus. The ﬁrst step consists of three PCRs: two to amplify the upstream and downstream regions of the target gene and one to amplify the selectable marker. The internal PCR primers have complementary tails used in the second round of PCRs for mutually primed synthesis. The result is a disruption fragment with long regions of homology to the upstream and downstream ﬂanking sequences of the target gene (350 nucleotides in this example). After the selection of transformants on the appropriate single or double amino acid dropout medium, gene disruption candidates were screened by PCR for expected 5’ and 3’ junctions as well as the size of the disrupted gene.

**1.5 Ectopic overexpression of target genes**

The target genes were first introduced into the pCPC18 vector for ectopic overexpression.^[3]^ The first round of PCR used F1 and R1 primers to amplify the target gene, generating a product with a 15-bp flank homology region. Then, these two products were assembled via ligation-independent cloning. After transformation and integration, the target gene was integrated into the *ADE2* locus and controlled by the constitutive *ADH1* promoter.

**1.6 Molecular docking and molecular dynamics simulations**

The predicted structure of Sec31 was generated using AlphaFold. The protonation states of small molecules were set at pH = 7.4, and compounds were expanded to 3D structures using Open Babel.^[4]^ A series of preparations for Sec31 and OTB were carried out using the AutoDock Tools (ADT3). Docking grids were generated through the AutoGrid program, followed by molecular docking using AutoDock Vina (1.2.0).^[5, 6]^ The optimal binding conformations were selected for interaction analysis. Finally, Sec31-OTB interaction diagrams were generated using PyMOL. Sec31 is represented as a deep blue cartoon model, the ligand is depicted as a cyan stick model, and their binding sites are illustrated as magenta stick structures. Non-polar hydrogen atoms are omitted. Hydrogen bonds, ionic interactions, and hydrophobic interactions are depicted as yellow, magenta, and green dashed lines, respectively.

For molecular dynamics simulations, the molecular dynamics simulations were carried out with Desmond/Maestro noncommercial version 2022.1 as molecular dynamics software.^[7, 8]^ TIP3P water molecules were added to the systems, which were then neutralized by 0.15 M NaCl solution. After minimization and relaxation of the system, the production simulation was performed for 100 ns in an isothermal-isobaric ensemble at 300 K and 1 bar. Trajectory coordinates were recorded every 100 ps. The molecular dynamics analysis was performed using a Simulation Interaction Diagram from Desmond.

**1.7 Extraction and mass spectrometry analysis of sterols**

As previously mentioned, the extraction and analysis of sterols were conducted as described in a previous study.^[9]^ *C. albicans* cells were collected by centrifugation at 4,500 g for 3 min at 4°C and washed three times with 10 mL of sterile double-distilled H_2_O (ddH_2_O). The resulting pellets were weighed, and their wet weight was adjusted to a range of 0.5-0.7 g. The cell pellets were thoroughly mixed with 2.5 mL of sterile ddH_2_O and 6 mL of 15% (W/V) NaOH (soluble in 90% [V/V] ethanol; Sangon biotech, A100173) in a 50 mL culture tube. The mixture was then incubated at 80 ℃ in a water bath for 1 hour, with vortexing performed every 20 min. Following the incubation period, the samples could cool to room temperature. Subsequently, 6 mL of petroleum ether (Sinopharm chemical reagent, 10015218) was added to each sample for extraction. The tubes were vigorously shaken by hand and left undisturbed for 1 min. The supernatant was collected once significant stratification was observed, and this process was repeated three times. Following the washing and subsequent drying at a water bath temperature of 65°C for approximately 30 min. The samples were then dissolved in 600 µL of n-hexane (Sangon biotech, A530007), with 400 µL being transferred into a 1.5 mL tube for derivatization. To serve as an internal standard, 8 µL of 5 mg/mL cholesterol (dissolved in n-hexane; Sangon biotech, A100433) was added, and the resulting mixture was frozen in a -20°C refrigerator. The icebox containing the samples was transported, and the percentage of sterols was determined through the analysis of gas chromatography-mass spectrometry (GC-MS). The sterol peak areas were standardized by normalizing them to the internal extraction efficiency control (cholesterol) and the wet weights of each sample. The assays were performed in triplicate and represent biological duplicates. The data were plotted using Prism v9.3.0 (GraphPad), displaying the mean relative quantity ± standard deviation (SD).

**1.8 Plasma membrane potential evaluation**

The plasma membrane potential changes were assessed using the membrane potential molecular probe, DiBAC4(3) (bis-(1,3-dibarbituric acid)-trimethine oxanol), as described before with some modification.^[10]^ Briefly, overnight *C. albicans* cultures were diluted to an OD600 of 0.2 and grown in the presence of the respective compounds (or DMSO equivalent) for 30 min at 30°C. Cells were then pelleted by centrifugation at 2,500 g for 3 min and washed three times in PBS. The membrane potential sensitive dye DiBAC4(3) (Med Chem Express, HY-101892) was added at a final concentration of 20 μg/ml in PBS, and the samples were incubated for 30 min at 37°C in the dark. The percentage of depolarized fluorescent *C. albicans* cells in the suspension was determined by flow cytometry.

**2. Supplementary figures**

**
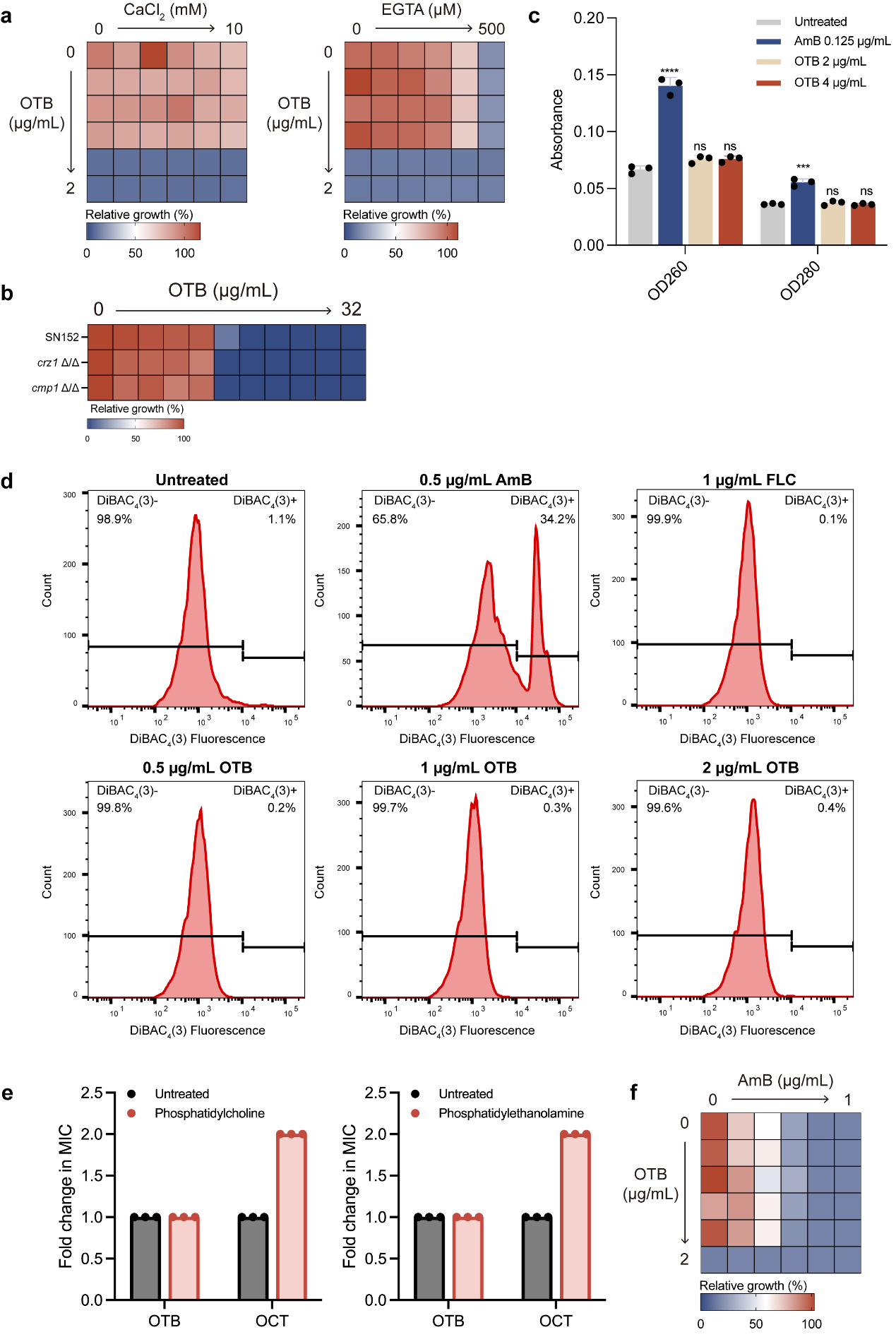
**

**Figure S1**. The mechanism by which OTB disrupts the cell membrane does not involve disrupting Ca^2+^ homeostasis. (**a**) (**Left**) Dose-matrix titration assays with CaCl_2_ in combination with OTB. (**Right**) Dose-matrix titration assays with EGTA in combination with OTB. (**b**) Broth microdilution assays were performed against wild-type and null mutant strains of *CRZ1* and *CMP1* in the presence of OTB. (**c**) Effects of OTB on the permeability and membrane integrity of *C. albicans* cells. *C. albicans* (SN152) cells were treated with the indicated condition for 4 h. The absorbance of the supernatant was determined at 260/280 nm using a UV–vis spectrophotometer. Data are presented as mean ± SD of technical triplicates. Significance was determined by one-way ANOVA with Bonferroni’s multiple comparisons test; *p*-value: ***<0.001, ****<0.0001. (**d**) The changes of cell membrane potential were assessed in OTB-treated C. albicans cells using DiBAC4 (3) staining, using AmB for comparison. (**e**) Change in the antifungal activity of OTB in the presence of phosphatidylcholine and phosphatidylethanolamine, using octenidine for comparison. (**f**) Dose-matrix titration assays with AmB in combination with OTB. **
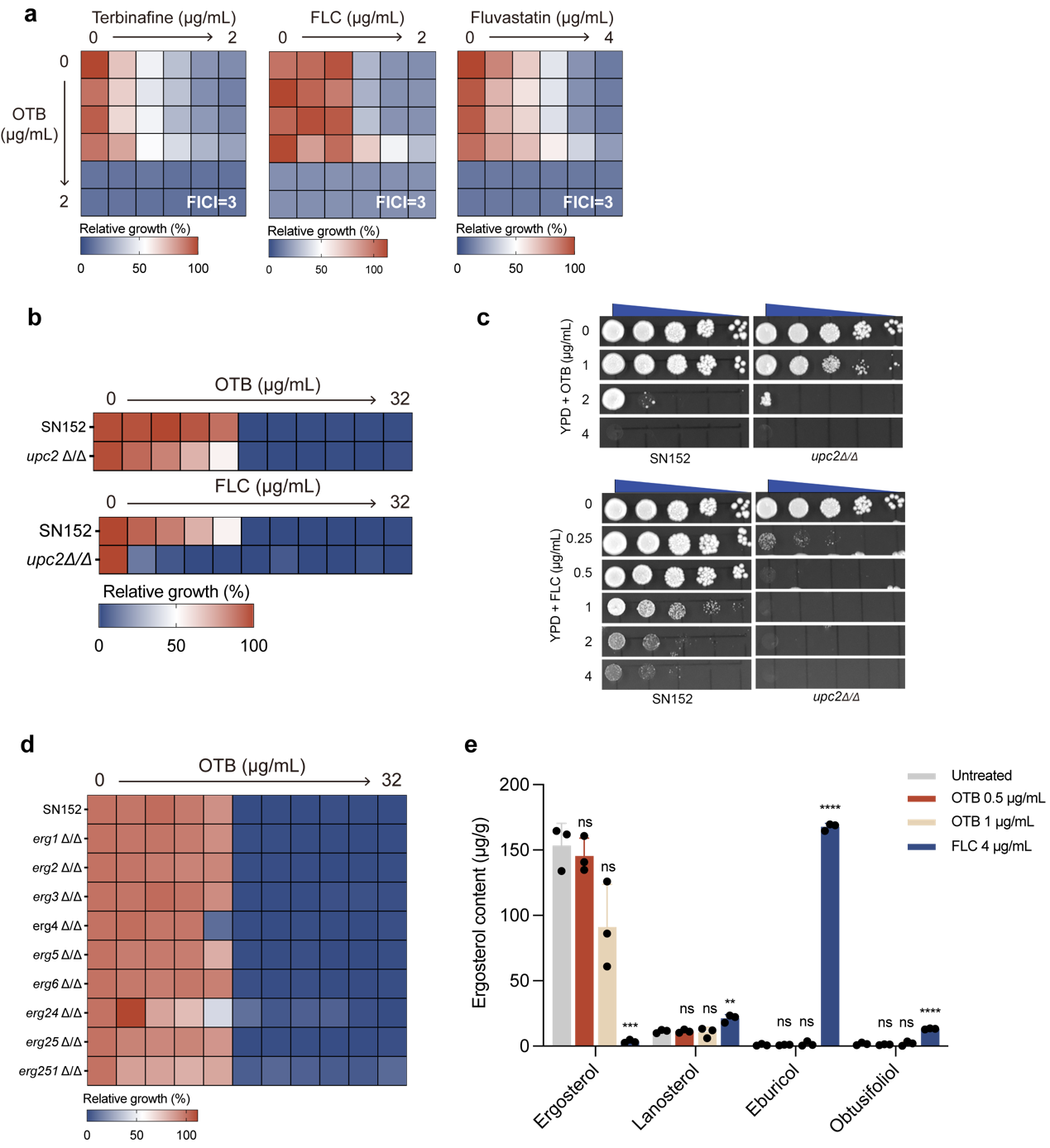
Figure S2**. OTB is not an ergosterol synthesis inhibitor. (**a**) Dose-matrix titration assays with terbinafine, fluconazole, and fluvastatin in combination with OTB. (**b**) Broth microdilution assays were performed against wild-type and null mutant strains of *UPC2* in the presence of OTB or FLC as described previously. (**c**) Spot assays of OTB and FLC were performed against wild-type and null mutant strains of *UPC2* separately. (**d**) Broth microdilution assays were performed against wild-type and null mutant strains of *ERG1*, *ERG2*, *ERG3*, *ERG4*, *ERG5*, *ERG6*, *ERG24*, *ERG25*, and *ERG251* in the presence of OTB. (**e**) The content of ergosterol, lanosterol, eburicol, and obtusifoliol per gram of fungal determined in *C. albicans* (SN152) after 1 μg/mL OTB treatment relative to internal cholesterol standard. Data are presented as mean ± SD of triplicate runs. Signiﬁcance was determined by one-way ANOVA with Bonferroni’s multiple comparisons test; *p*-value: *<0.05, **<0.01, ***<0.001, ****<0.0001.

**
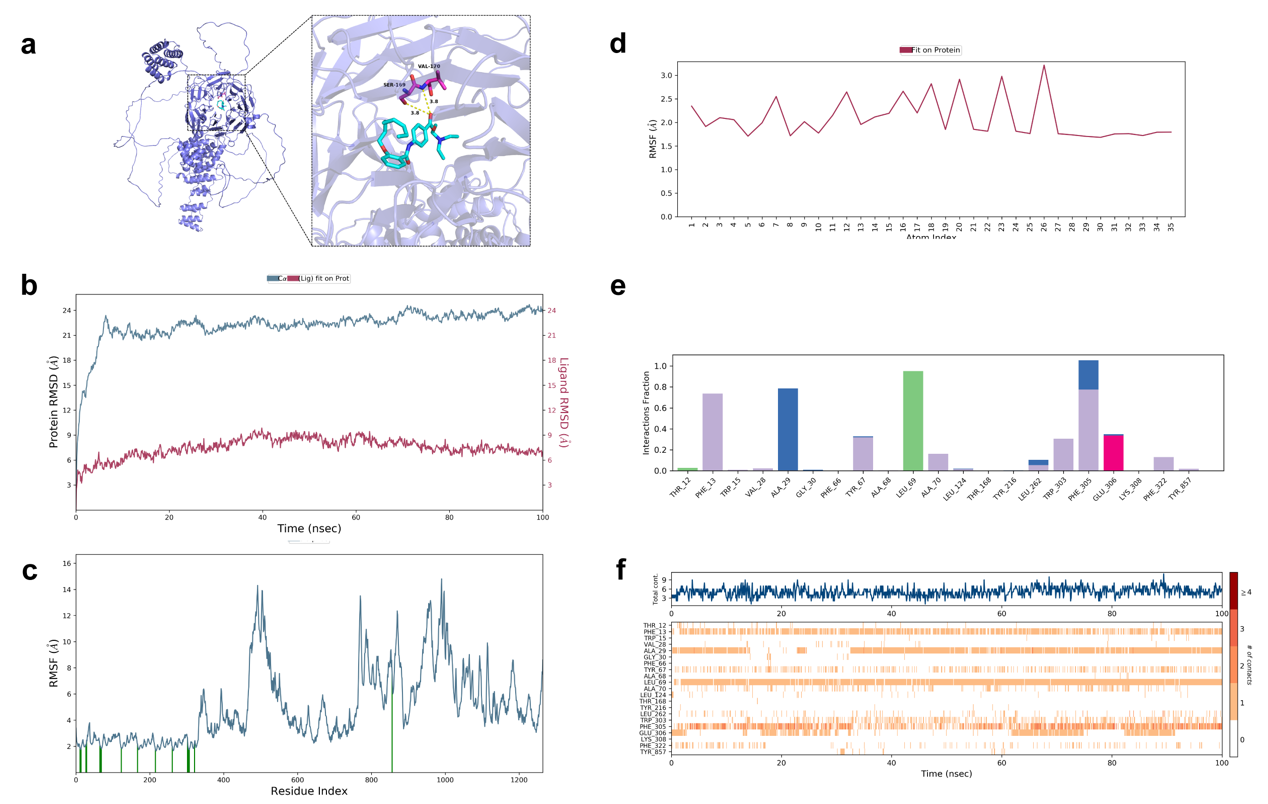
Figure S3.** Molecular dynamic simulation results after docking of OTB with Sec31. (**a**) The ligand-binding site of Sec31 with OTB is predicted by the Autodock Vina program. Sec31 is represented as a deep blue cartoon model, while OTB is depicted as a cyan stick model, and their binding sites are illustrated as magenta stick structures. Non-polar hydrogen atoms are omitted. Hydrogen bonds, ionic, and hydrophobic interactions are depicted as yellow, magenta, and green dashed lines, respectively. (**b**) Root mean square deviation (RMSD). (**c**) Root mean square fluctuation (RMSF). (**d**) Ligand root mean square fluctuation (L-RMSF). (**e**) Protein-ligand contacts simulation results after docking of OTB with the Sec31 protein. Various types of protein residue and ligand contacts: Hydrogen Bonds (green), Hydrophobic (purple), Ionic (red), and Water Bridges (blue). (**f**) Timeline representation of the interactions and contacts (H-bonds, Hydrophobic, Ionic, Water bridges) summarized in the (**e**).

**
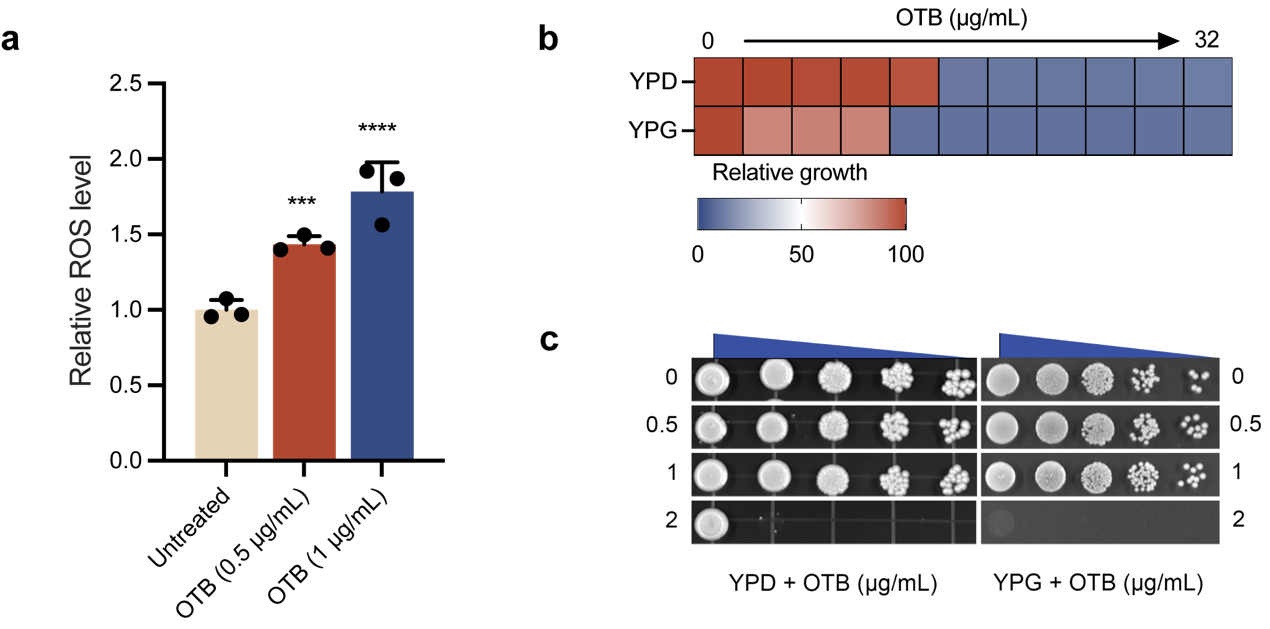
Figure S4.** OTB disrupts intracellular mitochondrial function. **(a**) The impact of OTB treatment on the ROS levels of *C. albicans* cells was assessed. Data are presented as mean ± SD of technical triplicates. Significance was determined using one-way ANOVA with Bonferroni’s multiple comparisons test; *p*-value: ***<0.001, ****<0.0001. (**b**) The broth microdilution assays were conducted in both YPD and YPG medium in the presence of OTB. (**c**) Spot assays of OTB against SN152 were performed in YPD and YPG medium, respectively.

**
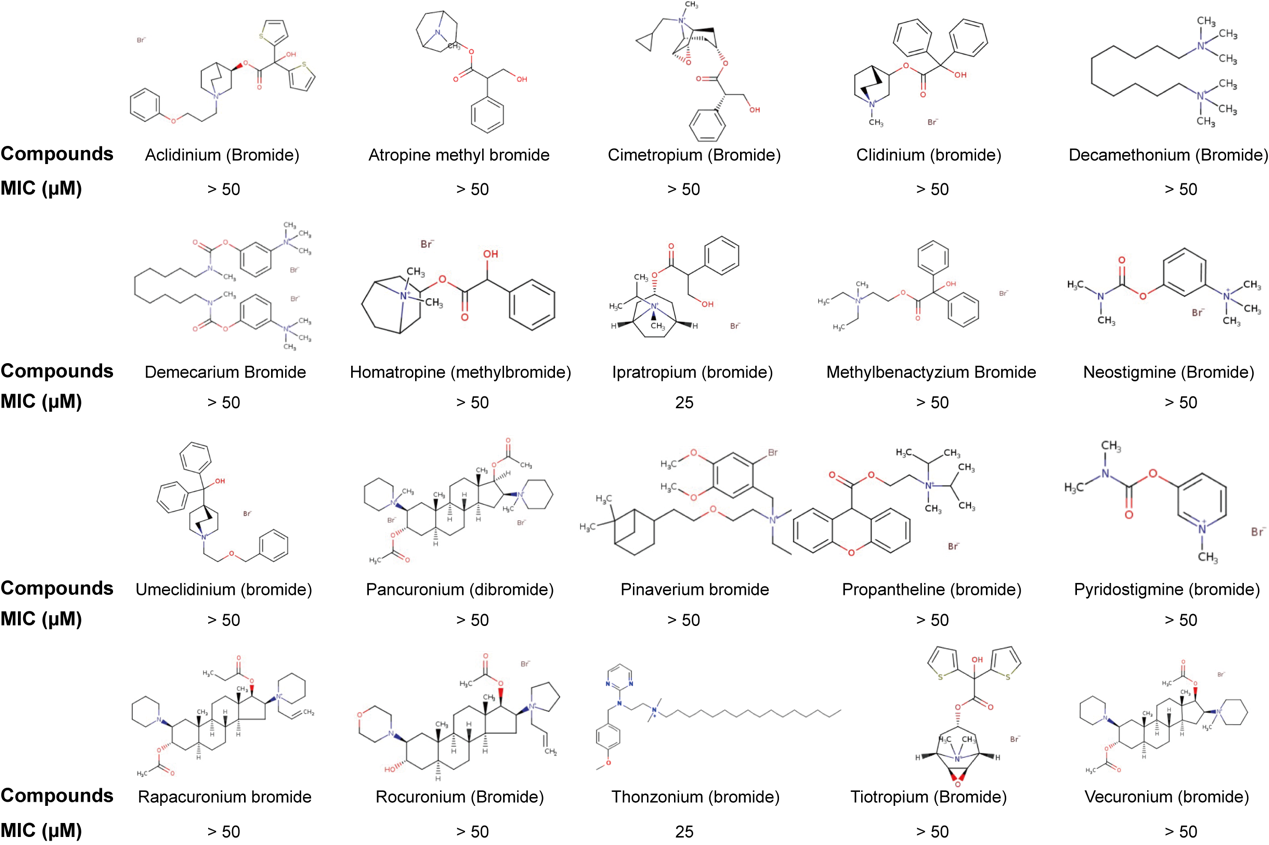
Figure S5.** The structure and antifungal activity of compounds containing quaternary ammonium salt groups.

**
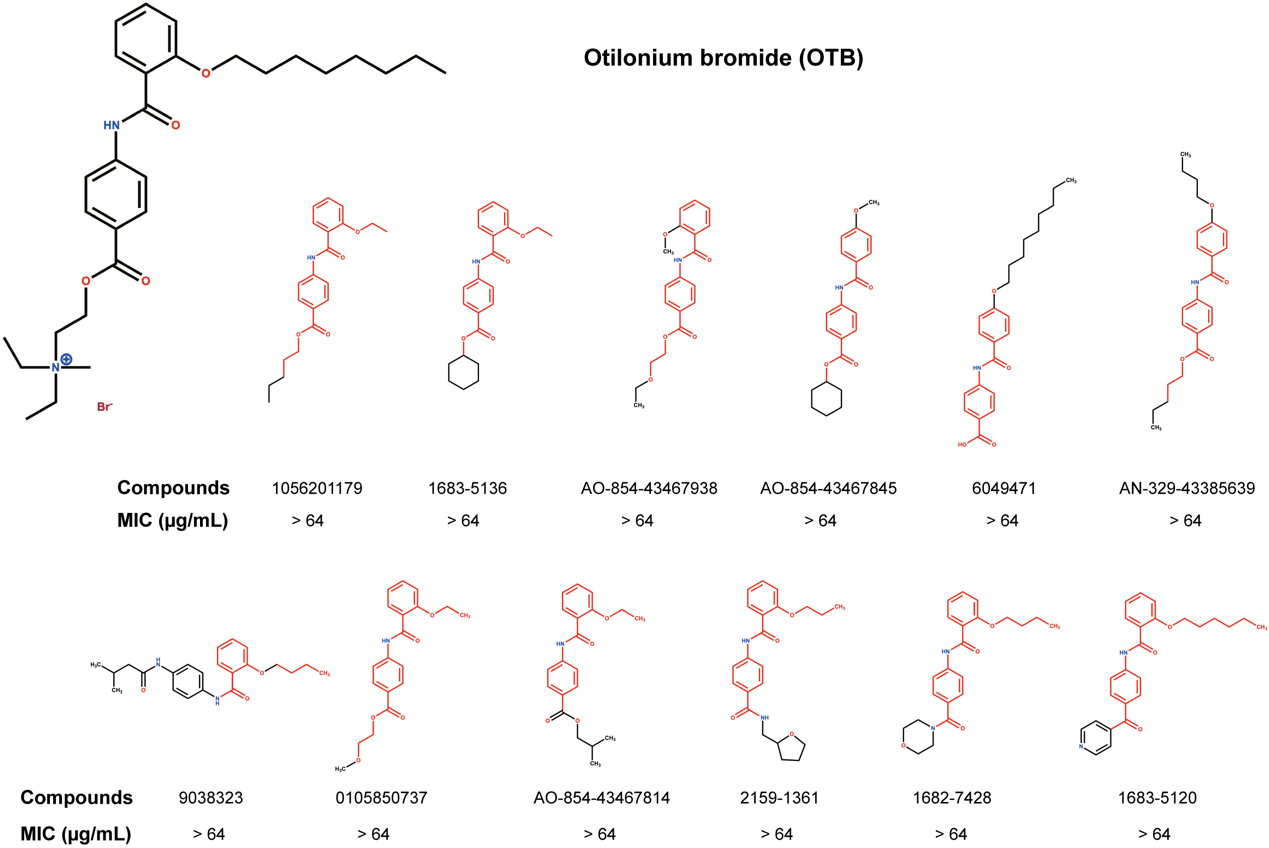
Figure S6.** The structure and antifungal activity of compounds structure like OTB.

**3. Supplementary tables**

**Table S1. Proteins (120-140 kDa) in band**

| **No.** | **Accession** | **Description** | **# Peptides** | **# PSMs** | **# Unique Peptides** | **# AAs** | | **MW [kDa]** | **# Razor Peptides** | | **Found in Sample: [S34] F34: Sample** |
| --- | --- | --- | --- | --- | --- | --- | --- | --- | --- | --- | --- |
| **1** | Q59RI1 | Isoleucyl-tRNA synthetase OS=Candida albicans (strain SC5314 / ATCC MYA-2876) OX=237561 GN=ILS1 PE=1 SV=1 | 83 | 194 | 4 | 1088 | 125.2 | | 88 | High | |
| **2** | A0A1D8PS12 | Leucyl-tRNA synthetase OS=Candida albicans (strain SC5314 / ATCC MYA-2876) OX=237561 GN=CDC60 PE=4 SV=1 | 74 | 131 | 24 | 1097 | 125.3 | | 57 | High | |
| **3** | A0A1D8PLY4 | Pyruvate carboxylase OS=Candida albicans (strain SC5314 / ATCC MYA-2876) OX=237561 GN=PYC2 PE=4 SV=1 | 69 | 120 | 69 | 1177 | 129.6 | | 0 | High | |
| **4** | Q5AF38 | Alpha-mannosidase OS=Candida albicans (strain SC5314 / ATCC MYA-2876) OX=237561 GN=AMS1 PE=3 SV=1 | 55 | 73 | 55 | 1155 | 131.5 | | 0 | High | |
| **5** | Q5AED0 | Glyco_hydro_63 domain-containing protein OS=Candida albicans (strain SC5314 / ATCC MYA-2876) OX=237561 GN=orf19.338 PE=3 SV=1 | 46 | 56 | 17 | 1047 | 121.2 | | 29 | High | |
| **6** | A0A1D8PHR0 | Valyl-tRNA synthetase OS=Candida albicans (strain SC5314 / ATCC MYA-2876) OX=237561 GN=VAS1 PE=3 SV=1 | 50 | 63 | 50 | 1119 | 127.5 | | 0 | High | |
| **7** | Q5A8A6 | Carbamoyl-phosphate synthase (Glutamine-hydrolyzing) OS=Candida albicans (strain SC5314 / ATCC MYA-2876) OX=237561 GN=CPA2 PE=4 SV=1 | 46 | 62 | 43 | 1149 | 127 | | 0 | High | |
| **8** | Q5A1D5 | FACT complex subunit SPT16 OS=Candida albicans (strain SC5314 / ATCC MYA-2876) OX=237561 GN=CDC68 PE=3 SV=2 | 44 | 55 | 1 | 1060 | 121.3 | | 0 | High | |
| **9** | A0A1D8PDL7 | Uncharacterized protein OS=Candida albicans (strain SC5314 / ATCC MYA-2876) OX=237561 GN=orf19.2489 PE=4 SV=1 | 34 | 52 | 34 | 1109 | 122.1 | | 0 | High | |
| **10** | A0A1D8PF90 | Uncharacterized protein OS=Candida albicans (strain SC5314 / ATCC MYA-2876) OX=237561 GN=orf19.5281 PE=4 SV=1 | 44 | 50 | 1 | 1220 | 133.9 | | 44 | High | |
| **11** | A0A1D8PIF6 | Sulfite reductase subunit alpha OS=Candida albicans (strain SC5314 / ATCC MYA-2876) OX=237561 GN=MET10 PE=3 SV=1 | 38 | 45 | 38 | 1094 | 121.9 | | 0 | High | |
| **12** | Q5A8Y5 | DNA-directed RNA polymerase subunit beta OS=Candida albicans (strain SC5314 / ATCC MYA-2876) OX=237561 GN=orf19.3349 PE=3 SV=1 | 33 | 42 | 33 | 1234 | 139.8 | | 0 | High | |
| **13** | Q5ADW3 | Cullin-associated NEDD8-dissociated protein 1 OS=Candida albicans (strain SC5314 / ATCC MYA-2876) OX=237561 GN=TIP120 PE=3 SV=2 | 36 | 37 | 36 | 1195 | 134.4 | | 0 | High | |
| **14** | Q59UE8 | Structural maintenance of chromosomes protein 5 OS=Candida albicans (strain SC5314 / ATCC MYA-2876) OX=237561 GN=SMC5 PE=3 SV=1 | 32 | 36 | 32 | 1073 | 124.3 | | 0 | High | |
| **15** | A0A1D8PQM5 | Uncharacterized protein OS=Candida albicans (strain SC5314 / ATCC MYA-2876) OX=237561 GN=orf19.7067 PE=4 SV=1 | 31 | 34 | 16 | 1091 | 123.7 | | 15 | High | |
| **16** | A0A1D8PJB0 | Coatomer subunit alpha OS=Candida albicans (strain SC5314 / ATCC MYA-2876) OX=237561 GN=orf19.1672 PE=4 SV=1 | 30 | 31 | 5 | 1221 | 138.1 | | 21 | High | |
| **17** | Q59M52 | DNA-directed RNA polymerase subunit beta OS=Candida albicans (strain SC5314 / ATCC MYA-2876) OX=237561 GN=CAALFM_CR05550CA PE=3 SV=1 | 31 | 34 | 31 | 1157 | 129.2 | | 0 | High | |
| **18** | A0A1D8PFL0 | Uncharacterized protein OS=Candida albicans (strain SC5314 / ATCC MYA-2876) OX=237561 GN=orf19.4929 PE=4 SV=1 | 29 | 30 | 4 | 1174 | 137.9 | | 22 | High | |
| **19** | A0A1D8PL54 | Uncharacterized protein OS=Candida albicans (strain SC5314 / ATCC MYA-2876) OX=237561 GN=orf19.4163 PE=4 SV=1 | 25 | 26 | 25 | 1072 | 123.4 | | 0 | High | |
| **20** | A0A1D8PGM4 | U3 small nucleolar RNA-associated protein 22 OS=Candida albicans (strain SC5314 / ATCC MYA-2876) OX=237561 GN=UTP22 PE=3 SV=1 | 25 | 27 | 25 | 1175 | 133.6 | | 0 | High | |
| **21** | A0A1D8PPF9 | Exocyst complex component Sec8 OS=Candida albicans (strain SC5314 / ATCC MYA-2876) OX=237561 GN=SEC8 PE=3 SV=1 | 21 | 23 | 21 | 1107 | 126.8 | | 0 | High | |
| **22** | Q5A7S5 | Pre-mRNA-splicing factor RSE1 OS=Candida albicans (strain SC5314 / ATCC MYA-2876) OX=237561 GN=RSE1 PE=3 SV=1 | 21 | 23 | 21 | 1219 | 136.3 | | 0 | High | |
| **23** | A0A1D8PTY4 | Elf1p OS=Candida albicans (strain SC5314 / ATCC MYA-2876) OX=237561 GN=ELF1 PE=3 SV=1 | 19 | 20 | 18 | 1195 | 133.8 | | 0 | High | |
| **24** | A0A1D8PI61 | Msu1p OS=Candida albicans (strain SC5314 / ATCC MYA-2876) OX=237561 GN=MSU1 PE=4 SV=1 | 17 | 17 | 17 | 1146 | 131.5 | | 0 | High | |
| **25** | Q5AAU3 | Protein transport protein SEC31 OS=Candida albicans (strain SC5314 / ATCC MYA-2876) OX=237561 GN=PGA63 PE=3 SV=2 | 13 | 13 | 13 | 1265 | 136.2 | | 0 | High | |
| **26** | Q5AND8 | Ubiquitin-ubiquitin ligase OS=Candida albicans (strain SC5314 / ATCC MYA-2876) OX=237561 GN=orf19.5965 PE=3 SV=1 | 15 | 15 | 15 | 1075 | 123.5 | | 0 | High | |
| **27** | Q59Q34 | Ion-transporting P-type ATPase OS=Candida albicans (strain SC5314 / ATCC MYA-2876) OX=237561 GN=SPF1 PE=3 SV=1 | 16 | 16 | 16 | 1223 | 136.9 | | 0 | High | |
| **28** | Q5A310 | ISWI chromatin-remodeling complex ATPase ISW2 OS=Candida albicans (strain SC5314 / ATCC MYA-2876) OX=237561 GN=ISW2 PE=2 SV=1 | 14 | 15 | 8 | 1056 | 123 | | 0 | High | |
| **29** | A0A1D8PI59 | Structural maintenance of chromosomes protein OS=Candida albicans (strain SC5314 / ATCC MYA-2876) OX=237561 GN=SMC2 PE=3 SV=1 | 20 | 20 | 20 | 1171 | 133.9 | | 0 | High | |
| **30** | Q59P41 | Uncharacterized protein OS=Candida albicans (strain SC5314 / ATCC MYA-2876) OX=237561 GN=orf19.5495 PE=4 SV=1 | 13 | 14 | 13 | 1088 | 122.1 | | 0 | High | |
| **31** | A0A1D8PJ95 | MMS19 nucleotide excision repair protein (Fragment) OS=Candida albicans (strain SC5314 / ATCC MYA-2876) OX=237561 GN=MET18 PE=3 SV=1 | 14 | 14 | 10 | 1084 | 125.1 | | 3 | High | |
| **32** | A0A1D8PRD5 | ATP-dependent RNA helicase OS=Candida albicans (strain SC5314 / ATCC MYA-2876) OX=237561 GN=orf19.1335 PE=3 SV=1 | 17 | 18 | 17 | 1062 | 120.5 | | 0 | High | |
| **33** | A0A1D8PQ25 | DNA-directed DNA polymerase OS=Candida albicans (strain SC5314 / ATCC MYA-2876) OX=237561 GN=POL5 PE=3 SV=1 | 14 | 14 | 14 | 1055 | 121.5 | | 0 | High | |
| **34** | A0A1D8PGW7 | Uncharacterized protein OS=Candida albicans (strain SC5314 / ATCC MYA-2876) OX=237561 GN=orf19.864 PE=4 SV=1 | 14 | 15 | 14 | 1034 | 120.3 | | 0 | High | |
| **35** | A0A1D8PEA9 | TOG domain-containing protein OS=Candida albicans (strain SC5314 / ATCC MYA-2876) OX=237561 GN=orf19.5085 PE=4 SV=1 | 9 | 11 | 9 | 1091 | 120.5 | | 0 | High | |
| **36** | A0A1D8PLF7 | Uncharacterized protein OS=Candida albicans (strain SC5314 / ATCC MYA-2876) OX=237561 GN=orf19.4627 PE=4 SV=1 | 10 | 10 | 10 | 1114 | 128 | | 0 | High | |
| **37** | A0A1D8PI01 | Condensin complex subunit 1 OS=Candida albicans (strain SC5314 / ATCC MYA-2876) OX=237561 GN=YCS4 PE=3 SV=1 | 12 | 13 | 12 | 1172 | 131.8 | | 0 | High | |
| **38** | A0A1D8PNK4 | Uncharacterized protein OS=Candida albicans (strain SC5314 / ATCC MYA-2876) OX=237561 GN=orf19.4325 PE=4 SV=1 | 9 | 9 | 1 | 1143 | 127.1 | | 7 | High | |
| **39** | Q59ZE6 | AAA family ATPase peroxin 6 OS=Candida albicans (strain SC5314 / ATCC MYA-2876) OX=237561 GN=PEX6 PE=4 SV=1 | 9 | 9 | 9 | 1157 | 130.3 | | 0 | High | |
| **40** | A0A1D8PIS7 | tRNA methylation protein OS=Candida albicans (strain SC5314 / ATCC MYA-2876) OX=237561 GN=orf19.5365 PE=4 SV=1 | 10 | 10 | 10 | 1210 | 138.7 | | 0 | High | |
| **41** | A0A1D8PRW9 | Boi2p OS=Candida albicans (strain SC5314 / ATCC MYA-2876) OX=237561 GN=BOI2 PE=4 SV=1 | 8 | 8 | 8 | 1172 | 126.3 | | 0 | High | |
| **42** | A0A1D8PEY6 | Tricalbin OS=Candida albicans (strain SC5314 / ATCC MYA-2876) OX=237561 GN=orf19.1840 PE=4 SV=1 | 7 | 7 | 7 | 1228 | 135.8 | | 0 | High | |
| **43** | Q5APK0 | PAN2-PAN3 deadenylation complex catalytic subunit PAN2 OS=Candida albicans (strain SC5314 / ATCC MYA-2876) OX=237561 GN=PAN2 PE=3 SV=2 | 7 | 7 | 7 | 1190 | 136.4 | | 0 | High | |
| **44** | A0A1D8PEK0 | Uncharacterized protein OS=Candida albicans (strain SC5314 / ATCC MYA-2876) OX=237561 GN=orf19.4795 PE=4 SV=1 | 6 | 6 | 6 | 1166 | 135 | | 0 | High | |
| **45** | A0A1D8PDN2 | Glutamine amidotransferase subunit OS=Candida albicans (strain SC5314 / ATCC MYA-2876) OX=237561 GN=orf19.2455 PE=3 SV=1 | 9 | 9 | 9 | 1131 | 126.9 | | 0 | High | |
| **46** | Q5AFD2 | mRNA-binding U2 snRNP complex subunit OS=Candida albicans (strain SC5314 / ATCC MYA-2876) OX=237561 GN=orf19.2675 PE=3 SV=1 | 7 | 7 | 7 | 1094 | 125 | | 0 | High | |
| **47** | A0A1D8PFK5 | Chitin synthase OS=Candida albicans (strain SC5314 / ATCC MYA-2876) OX=237561 GN=CHS3 PE=4 SV=1 | 4 | 4 | 4 | 1213 | 136.1 | | 0 | High | |
| **48** | A0A1D8PRN8 | Non-specific serine/threonine protein kinase OS=Candida albicans (strain SC5314 / ATCC MYA-2876) OX=237561 GN=KIN2 PE=4 SV=1 | 4 | 4 | 4 | 1212 | 133.8 | | 0 | High | |
| **49** | A0A1D8PL46 | Translocase OS=Candida albicans (strain SC5314 / ATCC MYA-2876) OX=237561 GN=orf19.5675 PE=3 SV=1 | 5 | 5 | 5 | 1102 | 125 | | 0 | High | |
| **50** | A0A1D8PL84 | PINc domain-containing protein OS=Candida albicans (strain SC5314 / ATCC MYA-2876) OX=237561 GN=orf19.4686 PE=4 SV=1 | 4 | 4 | 4 | 1200 | 137 | | 0 | High | |
| **51** | Q5ANI8 | RNA-binding ribosome biosynthesis protein OS=Candida albicans (strain SC5314 / ATCC MYA-2876) OX=237561 GN=MAK21 PE=3 SV=1 | 3 | 3 | 3 | 1066 | 121.6 | | 0 | High | |
| **52** | A0A1D8PDD2 | Phospholipid-transporting ATPase OS=Candida albicans (strain SC5314 / ATCC MYA-2876) OX=237561 GN=orf19.783 PE=3 SV=1 | 3 | 3 | 3 | 1163 | 130.6 | | 0 | High | |
| **53** | A0A1D8PJL3 | Cytokin_check_N domain-containing protein OS=Candida albicans (strain SC5314 / ATCC MYA-2876) OX=237561 GN=orf19.267 PE=4 SV=1 | 3 | 3 | 3 | 1213 | 130 | | 0 | High | |
| **54** | Q5AP97 | Mitosis inhibitor protein kinase SWE1 OS=Candida albicans (strain SC5314 / ATCC MYA-2876) OX=237561 GN=SWE1 PE=1 SV=2 | 1 | 1 | 1 | 1178 | 130.7 | | 0 | High | |
| **55** | A0A1D8PJ74 | Arf family guanine nucleotide exchange factor OS=Candida albicans (strain SC5314 / ATCC MYA-2876) OX=237561 GN=GEA2 PE=4 SV=1 | 2 | 2 | 2 | 1127 | 128 | | 0 | High | |
| **56** | A0A1D8PTM3 | DNA-dependent ATPase OS=Candida albicans (strain SC5314 / ATCC MYA-2876) OX=237561 GN=CAALFM_CR07910CA PE=4 SV=1 | 1 | 1 | 1 | 1055 | 121.1 | | 0 | High | |
| **57** | A0A1D8PKE5 | C2H2-type domain-containing protein OS=Candida albicans (strain SC5314 / ATCC MYA-2876) OX=237561 GN=orf19.7397 PE=4 SV=1 | 2 | 2 | 2 | 1089 | 123.5 | | 0 | High | |
| **58** | Q5ANL3 | Uncharacterized protein OS=Candida albicans (strain SC5314 / ATCC MYA-2876) OX=237561 GN=orf19.5890 PE=4 SV=1 | 1 | 1 | 1 | 1111 | 124.3 | | 0 | High | |
| **59** | A0A1D8PQS3 | Uncharacterized protein OS=Candida albicans (strain SC5314 / ATCC MYA-2876) OX=237561 GN=orf19.7006 PE=4 SV=1 | 1 | 1 | 1 | 1094 | 120.8 | | 0 | High | |

**Table S2. Strains were used in this study**

| **No.** | **Alias** | **Name** | **Genotype** | **Source** |
| --- | --- | --- | --- | --- |
| **1** | *C. albicans* | SC5314 | Prototrophic | Eukaryot Cell. 2005 Feb;4(2):298-309. |
| **2** | *C. albicans* | ATCC MYA-2876 | Prototrophic | This study |
| **3** | *C. albicans* | ATCC MYA-24433 | Prototrophic | This study |
| **4** | *C. albicans* | ATCC MYA-90028 | Prototrophic | This study |
| **5** | *C. albicans* | ATCC MYA-44505 | Prototrophic | This study |
| **6** | *C. albicans* | ATCC MYA-64550 | Prototrophic | This study |
| **7** | *C. albicans* | ATCC MYA-64548 | Prototrophic | This study |
| **8** | *C. albicans* | ATCC MYA-62342 | Prototrophic | This study |
| **9** | *C. albicans* | *C. albicans* 1 | Prototrophic | This study |
| **10** | *C. albicans* | *C. albicans* 2 | Prototrophic | This study |
| **11** | *C. albicans* | *C. albicans* 3 | Prototrophic | This study |
| **12** | *C. albicans* | *C. albicans* 4 | Prototrophic | This study |
| **13** | *C. albicans* | *C. albicans* 5 | Prototrophic | This study |
| **14** | *C. albicans* | *C. albicans* 6 | Prototrophic | This study |
| **15** | *C. albicans* | *C. albicans* 7 | Prototrophic | This study |
| **16** | *C. albicans* | *C. albicans* 8 | Prototrophic | This study |
| **17** | *C. albicans* | *C. albicans* 10 | Prototrophic | This study |
| **18** | *C. albicans* | *C. albicans* 11 | Prototrophic | This study |
| **19** | *C. albicans* | SN152 | *his1 /his1 arg4*/*arg4* *leu2* /*leu2* | Eukaryot Cell. 2005 Feb;4(2):298-309. |
| **20** | *C. albicans* | *sip3*Δ/*sip3*Δ | *sip3*::*HIS1* /*sip3*::*ARG4*  *leu2* /*leu2* | This study |
| **21** | *C. albicans* | *osh2*Δ/*osh2*Δ | *osh2*::*HIS1* /*osh2*::*ARG4*  *leu2* /*leu2* | This study |
| **22** | *C. albicans* | *osh3*Δ/*osh3*Δ | *osh3*::*HIS1* /*osh3*::*ARG4*  *leu2* /*leu2* | This study |
| **23** | *C. albicans* | *osh4*Δ/*osh4*Δ | *osh4*::*HIS1* /*osh4*::*ARG4*  *leu2* /*leu2* | This study |
| **24** | *C. albicans* | *osh7*Δ/*osh7*Δ | *osh7*::*HIS1* /*osh7*::*ARG4*  *leu2* /*leu2* | This study |
| **25** | *C. albicans* | *ysp2*Δ/*ysp2*Δ | *ysp2*::*HIS1* /*ysp2*::*ARG4*  *leu2* /*leu2* | This study |
| **26** | *C. albicans* | GFP*-Sec31* | *his1 /his1 arg4/arg4 SEC31/sec31::* GFP*-SEC31-LEU2* | This study |
| **27** | *C. albicans* | *BET5/bet5*Δ | *bet5*::*HIS1* *arg4*/*arg4*  *leu2* /*leu2* | This study |
| **28** | *C. albicans* | *DOP1/dop1*Δ | *dop1*::*HIS1* *arg4/arg4*  *leu2* /*leu2* | This study |
| **29** | *C. albicans* | *SEC14/sec14*Δ | *sec14*::*HIS1* *arg4/arg4*  *leu2* /*leu2* | This study |
| **30** | *C. albicans* | *SEC31/sec31*Δ | *sec31*::*HIS1* *arg4/arg4*  *leu2* /*leu2* | This study |
| **31** | *C. albicans* | *SNU13/snu13*Δ | *sun13*::*HIS1* *arg4/arg4*  *leu2* /*leu2* | This study |
| **32** | *C. albicans* | GFP*-Sip3* | *his1 /his1 arg4/arg4 SIP3/sip3::* GFP*-SIP3-LEU2* | This study |
| **33** | *C. albicans* | GFP*-Atg8* | *his1 /his1 arg4/arg4 ATG8/atg8::* GFP*-ATG8-LEU2* | This study |
| **34** | *C. albicans* | GFP-Atg8::*sec31*Δ | *sec31::HIS1 arg4/arg4 ATG8/atg8::* GFP*-ATG8-LEU2* | This study |
| **35** | *C. albicans* | GFP-Atg8::*tetO*-*SEC31*::*sec31*Δ | *sec31::his1* *SEC31/sec31::tetO-SEC31-arg4 ATG8/atg8::* GFP*-ATG8-LEU2* | This study |
| **36** | *C. albicans* | *Cdr1-*GFP::Pma1-YFP | *his1 /his1*  *CDR1* /*cdr1*:: *CDR1*-GFP-*LEU2 PMA1* /*pma1*:: *PMA1*-YFP-*ARG4* | Front Pharmacol. 2022 Dec 21;13:1101553. |
| **37** | *C. albicans* | *cdr1*Δ/*cdr1*Δ | *cdr1*::*HIS1* /*cdr1*::*ARG4*  *leu2* /*leu2* | Front Pharmacol. 2022 Dec 21;13:1101553. |
| **38** | *C. albicans* | *upc2*Δ/*upc2*Δ | *upc2*::*HIS1* /*upc2*::*ARG4*  *leu2* /*leu2* | mBio. 2023 Feb 28;14(1):e0263922. |
| **39** | *C. albicans* | *tac1*Δ/*tac1*Δ | *tac1*::*HIS1* /*tac1*::*ARG4*  *leu2* /*leu2* | This study |
| **40** | *C. albicans* | P*_ADH1_-CDR1* | *his1 /his1 arg4*/*arg4* *ADE2* /*ade2*:: ADH1p-*CDR1*-*LEU2* | This study |
| **41** | *C. albicans* | P_ADH1_-*CDR2* | *his1 /his1 arg4*/*arg4* *ADE2* /*ade2*:: ADH1p-*CDR2*-*LEU2* | This study |
| **42** | *C. albicans* | P_ADH1_-*-MDR1* | *his1 /his1 arg4*/*arg4* *ADE2* /*ade2*:: ADH1p-*MDR1*-*LEU2* | This study |
| **43** | *C. albicans* | *crz1*Δ/*crz1*Δ | *cmp1*::*HIS1* /*cmp1*::*ARG4*  *leu2* /*leu2* | Front Pharmacol. 2022 Dec 21;13:1101553. |
| **44** | *C. albicans* | *cmp1*Δ/*cmp1*Δ | *crz1*::*HIS1* /*crz1*::*ARG4*  *leu2* /*leu2* | Front Pharmacol. 2022 Dec 21;13:1101553. |
| **45** | *C. albicans* | *erg1*Δ/*erg1*Δ | *erg1*::*HIS1* /*erg1*::*ARG4*  *leu2* /*leu2* | mBio. 2023 Feb 28;14(1):e0263922. |
| **46** | *C. albicans* | *erg2*Δ/*erg2*Δ | *erg2*::*HIS1* /*erg2*::*ARG4*  *leu2* /*leu2* | mBio. 2023 Feb 28;14(1):e0263923. |
| **47** | *C. albicans* | *erg3*Δ/*erg3*Δ | *erg3*::*HIS1* /*erg3*::*ARG4*  *leu2* /*leu2* | mBio. 2023 Feb 28;14(1):e0263924. |
| **48** | *C. albicans* | *erg4*Δ/*erg4*Δ | *erg4*::*HIS1* /*erg4*::*ARG4*  *leu2* /*leu2* | mBio. 2023 Feb 28;14(1):e0263925. |
| **49** | *C. albicans* | *erg5*Δ/*erg5*Δ | *erg5*::*HIS1* /*erg5*::*ARG4*  *leu2* /*leu2* | mBio. 2023 Feb 28;14(1):e0263926. |
| **50** | *C. albicans* | *erg6*Δ/*erg6*Δ | *erg6*::*HIS1* /*erg6*::*ARG4*  *leu2* /*leu2* | mBio. 2023 Feb 28;14(1):e0263927. |
| **51** | *C. albicans* | *erg24*Δ/*erg24*Δ | *erg24*::*HIS1* /*erg24*::*ARG4*  *leu2* /*leu2* | mBio. 2023 Feb 28;14(1):e0263928. |
| **52** | *C. albicans* | *erg25*Δ/*erg25*Δ | *erg25*::*HIS1* /*erg25*::*ARG4*  *leu2* /*leu2* | mBio. 2023 Feb 28;14(1):e0263929. |
| **53** | *C. albicans* | *erg251*Δ/*erg251*Δ | *erg251*::*HIS1* /*erg251*::*ARG4*  *leu2* /*leu2* | mBio. 2023 Feb 28;14(1):e0263930. |
| **54** | *S. cerevisiae* | S288C | Prototrophic | This study |
| **55** | *S. cerevisiae* | BY4741 | *MAT*a *his3*Δ1 *leu2*Δ0 *met15*Δ0 *ura3*Δ0 | Mar Drugs. 2019 Jan 14;17(1):54. |
| **56** | *S. cerevisiae* | *osh2*Δ | BY4741 *YDL019C*Δ::*KanMX* | Mar Drugs. 2019 Jan 14;17(1):55. |
| **57** | *S. cerevisiae* | *osh3*Δ | BY4741 *YHR073W*Δ::*KanMX* | Mar Drugs. 2019 Jan 14;17(1):56. |
| **58** | *S. cerevisiae* | *osh6*Δ | BY4741 *YKR003W*Δ::*KanMX* | Mar Drugs. 2019 Jan 14;17(1):57. |
| **59** | *S. cerevisiae* | *lam1*Δ | BY4741 *YHR155W*Δ::*KanMX* | Mar Drugs. 2019 Jan 14;17(1):58. |
| **60** | *S. cerevisiae* | *ysp2*Δ | BY4741 *YDR326C*Δ::*KanMX* | Mar Drugs. 2019 Jan 14;17(1):59. |
| **61** | *S. cerevisiae* | *sip3*Δ | BY4741 *YNL257C*Δ::*KanMX* | Mar Drugs. 2019 Jan 14;17(1):60. |
| **62** | *S. cerevisiae* | *lam4*Δ | BY4741 *YHR080C*Δ::*KanMX* | Mar Drugs. 2019 Jan 14;17(1):61. |
| **63** | *S. cerevisiae* | *lam5*Δ | BY4741 *YFL042C*Δ::*KanMX* | Mar Drugs. 2019 Jan 14;17(1):62. |
| **64** | *S. cerevisiae* | *lam6*Δ | BY4741 *YLR072W*Δ::*KanMX* | Mar Drugs. 2019 Jan 14;17(1):63. |
| **65** | *S. cerevisiae* | *sbh2*Δ | BY4741 *YER019C-A*Δ::*KanMX* | Mar Drugs. 2019 Jan 14;17(1):55. |
| **66** | *S. cerevisiae* | *sec22*Δ | BY4741 *YLR268W*Δ::*KanMX* | Mar Drugs. 2019 Jan 14;17(1):56. |
| **67** | *S. cerevisiae* | *sec28*Δ | BY4741 *YIL076W*Δ::*KanMX* | Mar Drugs. 2019 Jan 14;17(1):57. |
| **68** | *S. cerevisiae* | *sec66*Δ | BY4741 *YBR171W*Δ::*KanMX* | Mar Drugs. 2019 Jan 14;17(1):58. |
| **69** | *S. cerevisiae* | *sfh5*Δ | BY4741 *YJL145W*Δ::*KanMX* | Mar Drugs. 2019 Jan 14;17(1):59. |
| **70** | *S. cerevisiae* | *ssh1*Δ | BY4741 *YBR283C*Δ::*KanMX* | Mar Drugs. 2019 Jan 14;17(1):60. |
| **71** | *S. cerevisiae* | *sec72*Δ | BY4741 *YLR292C*Δ::*KanMX* | Mar Drugs. 2019 Jan 14;17(1):61. |
| **72** | *S. cerevisiae* | *vps27*Δ | BY4741 *YNR006W*Δ::*KanMX* | Mar Drugs. 2019 Jan 14;17(1):55. |
| **73** | *S. cerevisiae* | *hse1*Δ | BY4741 *YHL002W*Δ::*KanMX* | Mar Drugs. 2019 Jan 14;17(1):56. |
| **74** | *S. cerevisiae* | *vps23*Δ | BY4741 *YCL008C*Δ::*KanMX* | Mar Drugs. 2019 Jan 14;17(1):57. |
| **75** | *S. cerevisiae* | *vps28*Δ | BY4741 *YPL065W*Δ::*KanMX* | Mar Drugs. 2019 Jan 14;17(1):58. |
| **76** | *S. cerevisiae* | *vps37*Δ | BY4741 *YLR119W*Δ::*KanMX* | Mar Drugs. 2019 Jan 14;17(1):59. |
| **77** | *S. cerevisiae* | *mvb12*Δ | BY4741 *YGR206W*Δ::*KanMX* | Mar Drugs. 2019 Jan 14;17(1):60. |
| **78** | *S. cerevisiae* | *snf8*Δ | BY4741 *YPL002C*Δ::*KanMX* | Mar Drugs. 2019 Jan 14;17(1):61. |
| **79** | *S. cerevisiae* | *vps25*Δ | BY4741 *YJR102C*Δ::*KanMX* | Mar Drugs. 2019 Jan 14;17(1):55. |
| **80** | *S. cerevisiae* | *vps36*Δ | BY4741 *YLR417W*Δ::*KanMX* | Mar Drugs. 2019 Jan 14;17(1):56. |
| **81** | *S. cerevisiae* | *vps2*Δ | BY4741 *YKL002W*Δ::*KanMX* | Mar Drugs. 2019 Jan 14;17(1):57. |
| **82** | *S. cerevisiae* | *vps20*Δ | BY4741 *YMR077C*Δ::*KanMX* | Mar Drugs. 2019 Jan 14;17(1):58. |
| **83** | *S. cerevisiae* | *vps24*Δ | BY4741 *YKL041W*Δ::*KanMX* | Mar Drugs. 2019 Jan 14;17(1):59. |
| **84** | *S. cerevisiae* | *snf7*Δ | BY4741 *YLR025W*Δ::*KanMX* | Mar Drugs. 2019 Jan 14;17(1):60. |
| **85** | *S. cerevisiae* | *vps60*Δ | BY4741 *YDR486C*Δ::*KanMX* | Mar Drugs. 2019 Jan 14;17(1):61. |
| **86** | *S. cerevisiae* | *did2*Δ | BY4741 *YKR035W-A*Δ::*KanMX* | Mar Drugs. 2019 Jan 14;17(1):55. |
| **87** | *S. cerevisiae* | *vps4*Δ | BY4741 *YPR173C*Δ::*KanMX* | Mar Drugs. 2019 Jan 14;17(1):56. |
| **88** | *S. cerevisiae* | *bro1*Δ | BY4741 *YPL084W*Δ::*KanMX* | Mar Drugs. 2019 Jan 14;17(1):57. |
| **89** | *S. cerevisiae* | *atg1*Δ | BY4741 *YGL180W*Δ::*KanMX* | Mar Drugs. 2019 Jan 14;17(1):56. |
| **90** | *S. cerevisiae* | *atg11*Δ | BY4741 *YPR049C*Δ::*KanMX* | Mar Drugs. 2019 Jan 14;17(1):57. |
| **91** | *S. cerevisiae* | *atg13*Δ | BY4741 *YPR185W*Δ::*KanMX* | Mar Drugs. 2019 Jan 14;17(1):58. |
| **92** | *S. cerevisiae* | *atg17*Δ | BY4741 *YLR423C*Δ::*KanMX* | Mar Drugs. 2019 Jan 14;17(1):59. |
| **93** | *S. cerevisiae* | *atg29*Δ | BY4741 *YPL166W*Δ::*KanMX* | Mar Drugs. 2019 Jan 14;17(1):60. |
| **94** | *S. cerevisiae* | *atg31*Δ | BY4741 *YDR022C*Δ::*KanMX* | Mar Drugs. 2019 Jan 14;17(1):61. |
| **95** | *S. cerevisiae* | *atg8*Δ | BY4741 *YBL078C*Δ::*KanMX* | Mar Drugs. 2019 Jan 14;17(1):55. |
| **96** | *S. cerevisiae* | *atg9*Δ | BY4741 *YDL149W*Δ::*KanMX* | Mar Drugs. 2019 Jan 14;17(1):56. |
| **97** | *S. cerevisiae* | *atg18*Δ | BY4741 *YFR021W*Δ::*KanMX* | Mar Drugs. 2019 Jan 14;17(1):57. |
| **98** | *C. auris* | FY279 | Prototrophic | This study |
| **99** | *C. auris* | *C. auris* 276 | Prototrophic | This study |
| **100** | *C. auris* | *C. auris* 277 | Prototrophic | This study |
| **101** | *C. auris* | *C. auris* 278 | Prototrophic | This study |
| **102** | *C. auris* | *C. auris* 279 | Prototrophic | This study |
| **103** | *C. auris* | *C. auris* 280 | Prototrophic | This study |
| **104** | *C. parapsilosis* | #12108 | Prototrophic | Antimicrobial Agents and Chemotherapy, 2013. 57(10): p. 5026-5036. |
| **105** | *C. parapsilosis* | ATCC 90018 | Prototrophic | This study |
| **106** | *C. parapsilosis* | ATCC 22019 | Prototrophic | This study |
| **107** | *C. parapsilosis* | *C. parapsilosis* 70 | Prototrophic | This study |
| **108** | *C. parapsilosis* | *C. parapsilosis* 71 | Prototrophic | This study |
| **109** | *C. parapsilosis* | *C. parapsilosis* 72 | Prototrophic | This study |
| **110** | *C. parapsilosis* | *C. parapsilosis* 73 | Prototrophic | This study |
| **111** | *C. parapsilosis* | *C. parapsilosis* 74 | Prototrophic | This study |
| **112** | *C. parapsilosis* | *C. parapsilosis* 77 | Prototrophic | This study |
| **113** | *C. parapsilosis* | *C. parapsilosis* 79 | Prototrophic | This study |
| **114** | *C. parapsilosis* | *C. parapsilosis* 80 | Prototrophic | This study |
| **115** | *C. parapsilosis* | *C. parapsilosis* 81 | Prototrophic | This study |
| **116** | *C. parapsilosis* | *C. parapsilosis* 82 | Prototrophic | This study |
| **117** | *C. tropicalis* | #12584 | Prototrophic | This study |
| **118** | *C. tropicalis* | ATCC MYA-750 | Prototrophic | This study |
| **119** | *C. tropicalis* | *C. tropicalis* 51 | Prototrophic | This study |
| **120** | *C. tropicalis* | *C. tropicalis* 52 | Prototrophic | This study |
| **121** | *C. tropicalis* | *C. tropicalis* 53 | Prototrophic | This study |
| **122** | *C. tropicalis* | *C. tropicalis* 54 | Prototrophic | This study |
| **123** | *C. tropicalis* | *C. tropicalis* 55 | Prototrophic | This study |
| **124** | *C. tropicalis* | *C. tropicalis* 57 | Prototrophic | This study |
| **125** | *C. tropicalis* | *C. tropicalis* 58 | Prototrophic | This study |
| **126** | *C. tropicalis* | *C. tropicalis* 59 | Prototrophic | This study |
| **127** | *C. tropicalis* | *C. tropicalis* 60 | Prototrophic | This study |
| **128** | *C. tropicalis* | *C. tropicalis* 61 | Prototrophic | This study |
| **129** | *C. neoformans* | H99 | Prototrophic | Microbiology Spectrum, 2021. 9(2): p. e00723-21. |
| **130** | *C. glabrata* | ATCC 2001 | *C. glabrata* | Prototrophic |
| **131** | *C. glabrata* | *C. glabrata* 211 | Prototrophic | This study |
| **132** | *C. glabrata* | *C. glabrata* 212 | Prototrophic | This study |
| **133** | *C. glabrata* | *C. glabrata* 213 | Prototrophic | This study |
| **134** | *C. glabrata* | *C. glabrata* 214 | Prototrophic | This study |
| **135** | *C. glabrata* | *C. glabrata* 215 | Prototrophic | This study |
| **136** | *C. glabrata* | *C. glabrata* 216 | Prototrophic | This study |
| **137** | *C. glabrata* | *C. glabrata* 217 | Prototrophic | This study |
| **138** | *C. glabrata* | *C. glabrata* 218 | Prototrophic | This study |
| **139** | *C. glabrata* | *C. glabrata* 219 | Prototrophic | This study |
| **140** | *C. glabrata* | *C. glabrata* 220 | Prototrophic | This study |
| **141** | *C. guilliermondii* | *C. guilliermondii* 478 | Prototrophic | This study |
| **142** | *C. guilliermondii* | *C. guilliermondii* 479 | Prototrophic | This study |
| **143** | *C. guilliermondii* | *C. guilliermondii* 480 | Prototrophic | This study |
| **144** | *C. guilliermondii* | *C. guilliermondii* 481 | Prototrophic | This study |
| **145** | *C. guilliermondii* | *C. guilliermondii* 482 | Prototrophic | This study |
| **146** | *C. guilliermondii* | *C. guilliermondii* 483 | Prototrophic | This study |
| **147** | *C. guilliermondii* | *C. guilliermondii* 484 | Prototrophic | This study |
| **148** | *C. guilliermondii* | *C. guilliermondii* 485 | Prototrophic | This study |
| **149** | *C. guilliermondii* | *C. guilliermondii* 487 | Prototrophic | This study |
| **150** | *C. guilliermondii* | *C. guilliermondii* 488 | Prototrophic | This study |
| **151** | *C. krusei* | ATCC 6258 | Prototrophic | This study |
| **152** | *C. krusei* | *C. krusei* 68 | Prototrophic | This study |
| **153** | *C. krusei* | *C. krusei* 69 | Prototrophic | This study |
| **154** | *C. krusei* | *C. krusei* 266 | Prototrophic | This study |
| **155** | *C. krusei* | *C. krusei* 267 | Prototrophic | This study |
| **156** | *C. krusei* | *C. krusei* 268 | Prototrophic | This study |
| **157** | *C. krusei* | *C. krusei* 471 | Prototrophic | This study |
| **158** | *C. krusei* | *C. krusei* 472 | Prototrophic | This study |
| **159** | *C. krusei* | *C. krusei* 474 | Prototrophic | This study |
| **160** | *C. krusei* | *C. krusei* 475 | Prototrophic | This study |
| **161** | *C. krusei* | *C. krusei* 477 | Prototrophic | This study |

**Table S3. Primers were used in this study**

| **Primers for genes deletion** | | |
| --- | --- | --- |
| **No.** | **Primer name** | **Primer sequence (5'to3')** |
| **1** | SIP3 P1 | AAGCTTGTCTTTACGCAAGT |
| **2** | SIP3 P3 | cacggcgcgcctagcagcggAAAGATTACTTCAAGTTGAT |
| **3** | SIP3 P4 | gtcagcggccgcatccctgcCATTTATGTGTATTATGGAA |
| **4** | SIP3 P6 | AATCCATTACTTGGCACTTG |
| **5** | OSH2 P1 | GTTTTGGTCTGTTTGCCGTC |
| **6** | OSH2 P3 | cacggcgcgcctagcagcggGATTTTAGCTGTTTATTCTA |
| **7** | OSH2 P4 | gtcagcggccgcatccctgcACTGTTTTAATTGTTGATTG |
| **8** | OSH2 P6 | GGTCCGGAAAAAGGACCAGT |
| **9** | OSH3 P1 | GTGTTTTATCAGTTTAGAAG |
| **10** | OSH3 P3 | cacggcgcgcctagcagcggATTTCAAGTTGGTATGGGAA |
| **11** | OSH3 P4 | gtcagcggccgcatccctgcATTTTACTATTTCTTTGTCT |
| **12** | OSH3 P6 | AATTGGGTTATGCTGAACTT |
| **13** | OSH4 P1 | TTTACTTTCCCGTTTCCAAT |
| **14** | OSH4 P3 | cacggcgcgcctagcagcggGACAAAAAAATATACTGTTG |
| **15** | OSH4 P4 | gtcagcggccgcatccctgcATGAATTTAGAGTAGGTTTT |
| **16** | OSH4 P6 | TGCAGAACTCTATGGAATGA |
| **17** | OSH7 P1 | GACCATATTTTACCCGGCAT |
| **18** | OSH7 P3 | cacggcgcgcctagcagcggGTACTGAATACAGATAAAAA |
| **19** | OSH7 P4 | gtcagcggccgcatccctgcTTTGATATATTGCAATTTAA |
| **20** | OSH7 P6 | AGCGACAAAAAATCGCCTAC |
| **21** | YSP2 P1 | GCAATTTAAAGGAACAACCG |
| **22** | YSP2 P3 | cacggcgcgcctagcagcggTTTTCCGTCTCCCGTGTTTT |
| **23** | YSP2 P4 | gtcagcggccgcatccctgcATTAGTTTGTAGTTGTGTAT |
| **24** | YSP2 P6 | TTCTGGGGCTTGATTTTAGA |
| **25** | BET5 P1 | CTTTTGCGGAGCTCAACTTA |
| **26** | BET5 P3 | cacggcgcgcctagcagcggAGGGCGTCTATATGATAGCA |
| **27** | BET5 P4 | gtcagcggccgcatccctgcAAAAATAAGCTTTAAAAACA |
| **28** | BET5 P6 | TTCAGTTGCTCTTGAGTGTC |
| **29** | DOP1 P1 | AGCATTTTGGCCACGACTGT |
| **30** | DOP1 P3 | cacggcgcgcctagcagcggCTTTTCCGATAAGTCGTCTT |
| **31** | DOP1 P4 | gtcagcggccgcatccctgcTGGATGAAAAGATGCCTTTA |
| **32** | DOP1 P6 | TTCAGTGTTCGTTGTGGAGT |
| **33** | SEC14 P1 | TGCCTTGTTCTTTTCACACA |
| **34** | SEC14 P3 | cacggcgcgcctagcagcggTTAGTGGGATGGGATGAGAA |
| **35** | SEC14 P4 | gtcagcggccgcatccctgcTCATATACACGTACACACAC |
| **36** | SEC14 P6 | CAGTTTAATAAACCCAACAG |
| **37** | SEC31 P1 | TTTCTTGATGTGTTGGTCAA |
| **38** | SEC31 P3 | cacggcgcgcctagcagcggCTTTTATGAATATTTTTCTT |
| **39** | SEC31 P4 | gtcagcggccgcatccctgcGAGTAAGAGTTTAAAATCAA |
| **40** | SEC31 P6 | GCAAAACTAGCTGAAGTTGT |
| **41** | SNU13 P1 | TTTTTTCTCTCCTCTCCCTT |
| **42** | SNU13 P3 | cacggcgcgcctagcagcggTTGTAAATAATTTAGTTCAG |
| **43** | SNU13 P4 | gtcagcggccgcatccctgcGTAGTAAATGCAATTCAAAC |
| **44** | SNU13 P6 | ATAGGGATCTTGATAGTGGT |
| **45** | TAC1 P1 | CTTCTCAGTTGGTTGGTATA |
| **46** | TAC1 P3 | cacggcgcgcctagcagcggGAGAGAAGGAGAAAGGCTCT |
| **47** | TAC1 P4 | gtcagcggccgcatccctgcGAGTTGTAATTGGTGAAAGC |
| **48** | TAC1 P6 | GCCCCTGGTGAAATTCCGAA |
| **49** | Universal primer 2 | ccgctgctaggcgcgccgtgACCAGTGTGATGGATATCTGC |
| **50** | Universal primer 5 | gcagggatgcggccgctgacAGCTCGGATCCACTAGTAACG |
| **Primers for diagnosising the genes null mutants** | | |
| **No.** | **Primer name** | **Primer sequence (5'to3')** |
| **1** | SIP3 Ucheck | CGCAAATCATGTATAAACCT |
| **2** | SIP3 Dcheck | TTTCGGGTATCAGAGCTAAT |
| **3** | OSH2 Ucheck | TTTGACTGACTCACAGAAGT |
| **4** | OSH2 Dcheck | AAACTCTTCTCATAGTGCAG |
| **5** | OSH3 Ucheck | CAACAAGAATTTTTGCTTGT |
| **6** | OSH3 Dcheck | TAATTGGGATGGTAAAAGTG |
| **7** | OSH4 Ucheck | AGTGATGTCTAACAAATGGT |
| **8** | OSH4 Dcheck | TGATTCAGCCATTGTTAATA |
| **9** | OSH7 Ucheck | TTCGTTTTCCTTAGGAGTAG |
| **10** | OSH7 Dcheck | CGCATGGTCTGAATGTAATT |
| **11** | YSP2 Ucheck | ATCATTTATGTAGCAAACAA |
| **12** | YSP2 Dcheck | GTTATACTTCTTTGGGGATT |
| **13** | BET5 upcheck | CGGAAGTATGTTATCTAAGG |
| **14** | BET5 dncheck | CGGCATCCCGTTCAATTTTT |
| **15** | DOP1 upcheck | GGGTCGATTGGAAGTCTAAT |
| **16** | DOP1 dncheck | GGGCATTAGATATAGCAGGT |
| **17** | SEC14 upcheck | ATTACTACAACACATATCCG |
| **18** | SEC14 dncheck | GTACCACAACAGATGAACAA |
| **19** | SEC31 upcheck | TGTGTGTGTATCCTGTTACA |
| **20** | SEC31 dncheck | AAACCTGGAAATACTCTTCG |
| **21** | SNU13 upcheck | CAAGTGGGCGTTCATTGTAA |
| **22** | SNU13 dncheck | AATCTCTGTGATGGAGGTGT |
| **23** | TAC1 Ucheck | GCGCTTCCAAATCAGTAATA |
| **24** | TAC1 Dcheck | CAAACTCAACTGCCAAATGG |
| **25** | HIS1 Left | ATTAGATACGTTGGTGGTTC |
| **26** | HIS1 Right | AACACAACTGCACAATCTGG |
| **27** | ARG4 Left | ACACAGAGATACCTTGTACT |
| **28** | ARG4 Right | ACGGAGTACCACATACGATG |
|  |  |  |
| **Primers for ectopic overexpression of genes** | | |
| **No.** | **Primer name** | **Primer sequence (5'to3')** |
| **1** | CDR1 OverF1 | TGTACTTAATAATTTCTTTAAAAGGTCAAAAACGAAAAATGCCACCTGACGTCTAAGAA |
| **2** | CDR1 OverR1 | TTTAGATTCATCTTGCGACGACATCTTAGAATCTGACATCGGTATGACCATGATTACCT |
| **3** | CDR1 OverF2 | TTTATTCTTTTATATCACATATATATTCTATTTATTTTTTGTACTTAATAATTTCTTTA |
| **4** | CDR1 OverR2 | GTTTTCTGAAGAAGAGTCTTGACTAATTGCCTTTTCTAATTTAGATTCATCTTGCGACG |
| **5** | CDR2 OverF1 | CAAATATATTTCACTAATTAACACATACAATAAAAACATTGCCACCTGACGTCTAAGAA |
| **6** | CDR2 OverR1 | GTCTAGCTGTTGCGACAAAGACGTGTTTGCAGTACTCATCGGTATGACCATGATTACCT |
| **7** | CDR2 OverF2 | CAATTGATATAGTTTTATTTATTTATTTATTTATTAATTCAAATATATTTCACTAATTA |
| **8** | CDR2 OverR2 | AACTGATGAATTGTCCAGTGCATCCACCCATGGCTTTTCGTCTAGCTGTTGCGACAAAG |
| **9** | MDR1 OverF1 | ATTGCCCCAATAGCAATACATATACTTACATAGAACTTCTGCCACCTGACGTCTAAGAA |
| **10** | MDR1 OverR1 | TCTACCAACAAAACTATCTCTCAAAAATCTGTAATGCATCGGTATGACCATGATTACCT |
| **11** | MDR1 OverF2 | ACTTTTTTTTATTCCGTAACAATCATATTATAATTTTACATTGCCCCAATAGCAATACA |
| **12** | MDR1 OverR2 | ATGGGCAAAGTATTTATGTTTTGATAAATGATAAGTCACTCTACCAACAAAACTATCTC |
| **13** | SEC31 OverF1 | TATTGTTTTGAAATTAGTAAAGAAAAATATTCATAAAAGTGCCACCTGACGTCTAAGAA |
| **14** | SEC31 OverF2 | CTATTTTTTTTTTCTTTGCCCCATTCTTTTGTTTTAAAGTATTGTTTTGAAATTAGTAA |
| **15** | SEC31 OverR1 | AAATGTTGAAGTACGTGCTATTTCACTAATCTTCACCATCGGTATGACCATGATTACCT |
| **16** | SEC31 OverR2 | ACCGGCTGCTAATAAGGGTAAATTTTTCAGACTCCAAGCAAATGTTGAAGTACGTGCTA |
| **17** | CaP19 | gctgtagtgccattgCATTGTTCACCACAAATGTTTCT |
| **18** | CaP28 | tgacaatgattacctATGGTAGCGATGCACGGT |
| **19** | VP18 | ATCCACTGTGCTCCGAAAAC |
| **20** | VP26 | TGCCTTGGGTGGCTATTTTA |
| **21** | CaP22 | TTGGACGCAGCCAAATCAC |
| **22** | CaP23 | TGAGTTCAAATGGCACACCAA |
| **23** | VP28 | ACCCCAAACATTTTGACTCG |
| **24** | VP29 | TCAGTTGATGTCCATGTTGTCA |
| **25** | VP30 | CGCAGGAAAGAACATGTGAG |
| **26** | VP31 | AACTGGAACACCAATGACAGG |
| **Primers for diagnosising the genes ectopic overexpression** | | |
| **No.** | **Primer name** | **Primer sequence (5'to3')** |
| **1** | CDR1 Overupcheck | CTTCATGTTCATATGGATTG |
| **2** | CDR1 Overdncheck | CGAATCATTACGATATAGAC |
| **3** | CDR2 Overupcheck | TGAAATTTTCGGAATCTGGG |
| **4** | CDR2 Overdncheck | GACAAGATGTGACCAGGTAG |
| **5** | MDR1 Overupcheck | TTCGGGATCATCATCACCAT |
| **6** | MDR1 Overdncheck | ACAGCCTGTCGCCATCGCCG |
| **7** | SEC31 Overupcheck | TTTCTTGATGTGTTGGTCAA |
| **8** | SEC31 Overdncheck | GCAAGGTCTTTTGTTTTAAT |
| **9** | VP16 | AATGCCAATACTCCTCTTTCTC |
| **10** | VP17 | TCAACAAACTCTTTCAACTTCTCA |
| **Primers for N-terminal tagging of genes** | | |
| **No.** | **Primer name** | **Primer sequence (5'to3')** |
| **1** | GFP-Sec31 F1 | TATTGTTTTGAAATTAGTAAAGAAAAATATTCATAAAAGTGCCACCTGACGTCTAAGAA |
| **2** | GFP-Sec31 R1 | AAATGTTGAAGTACGTGCTATTTCACTAATCTTCACCATCTGCGTTATCCTCGAGTTCT |
| **3** | GFP-Sec31 OverF2 | CTATTTTTTTTTTCTTTGCCCCATTCTTTTGTTTTAAAGTATTGTTTTGAAATTAGTAA |
| **4** | GFP-Sec31 OverR2 | ACCGGCTGCTAATAAGGGTAAATTTTTCAGACTCCAAGCAAATGTTGAAGTACGTGCTA |
| **5** | GFP-Sip3 F1 | AACAACAATTTTCATCAACTTGAAGTAATCTTTGCGAACTGCCACCTGACGTCTAAGAA |
| **6** | GFP-Sip3 R1 | TGGATCAGGCCCACTTTCAACTGATGAAGTCAGTGGCATCTGCGTTATCCTCGAGTTCT |
| **7** | GFP-Sip3 F2 | CCGTTAAACAACAACAGTTATAATTGAACATACAGTAAGAACAACAATTTTCATCAACT |
| **8** | GFP-Sip3 R2 | GTCGGATTTCGGCAGCTTCACCATTGATGAAAGTATATTTGGATCAGGCCCACTTTCAA |
| **9** | GFP-Atg8 F1 | TGGAAATAATACTAACAGAACTTAGAAACATCACTCATATGCCACCTGACGTCTAAGAA |
| **10** | GFP-Atg8 F2 | CACTCAATCTTTAATTGACCATCGACCACAAAAATAATATGGAAATAATACTAACAGAA |
| **11** | GFP-Atg8 R1 | CATACCAAAAGGATGCTCGTCTTTGAATTGTGATCTCATCTGCGTTATCCTCGAGTTCT |
| **12** | GFP-Atg8 R2 | GATCGGAAGAAGATTCAGAGGACTAAAAATCATGTAATACATACCAAAAGGATGCTCGT |
| **13** | VP42 | GCGGCCGTTACTAGTGGAT |
| **14** | VP43 | TCCCTGGTCTTATCTTCTCCAG |
| **Primers for diagnosising the genes N-terminal tagging** | | |
| **No.** | **Primer name** | **Primer sequence (5'to3')** |
| **1** | GFP-Atg8 upcheck | ATATACATCCCCATCGATTC |
| **2** | GFP-Atg8 dncheck | TTATCAATTTCGGGGATATC |
| **3** | GFP-Sec31 upcheck | TTTCTTGATGTGTTGGTCAA |
| **4** | GFP-Sec31 dncheck | GCAAGGTCTTTTGTTTTAAT |
| **5** | GFP-Sip3 upcheck | AAGCTTGTCTTTACGCAAGT |
| **6** | GFP-Sip3 dncheck | AATGAACTAGCCAAGGCTGT |

**Table S4. Compounds were used in this study**

| **No.** | **Compounds** | **Company** | **Solvent** | **Sotck** |
| --- | --- | --- | --- | --- |
| **1** | Otilonium bromide | MCE, Shanghai, China | DMSO | 10 mM at -20℃ |
| **2** | Dioscin | MCE, Shanghai, China | DMSO | 10 mM at -20℃ |
| **3** | Cetylpyridinium (chloride monohydrate) | MCE, Shanghai, China | DMSO | 10 mM at -20℃ |
| **4** | Cetylpyridinium (chloride) | MCE, Shanghai, China | DMSO | 10 mM at -20℃ |
| **5** | Clioquinol | MCE, Shanghai, China | DMSO | 10 mM at -20℃ |
| **6** | Fingolimod | MCE, Shanghai, China | DMSO | 10 mM at -20℃ |
| **7** | Fingolimod (hydrochloride) | MCE, Shanghai, China | DMSO | 10 mM at -20℃ |
| **8** | Chloroxine | MCE, Shanghai, China | DMSO | 10 mM at -20℃ |
| **9** | Triclosan | MCE, Shanghai, China | DMSO | 10 mM at -20℃ |
| **10** | Auranofin | MCE, Shanghai, China | DMSO | 10 mM at -20℃ |
| **11** | XTT salt [2,3-bis(2-methoxy-4-nitro-5-sulfophenyl)2H-tetrazolium-5-carboxanilide sodium salt] | Macklin, Shanghai, China | PBS | 0.5 mg/mL at -20℃ |
| **12** | Fluconazole | Aladdin, Shanghai, China | DMSO | 10 mg/mL at -20℃ |
| **13** | Ampohtericin B | Aladdin, Shanghai, China | DMSO | 6.4 mg/mL at -20℃ |
| **14** | Ergosterol | Sangon biotech, Shanghai, China | Absolute ethanol, Tween 80 | 10 mM at -20℃ |
| **15** | Filipin complex | MCE, Shanghai, China | DMSO | 10 mM at -20℃ |
| **16** | Thermolysin | Sigma, America | 1× TNC buffer | 10 mg/mL at -20℃ |
| **17** | FM4-64 | MCE, Shanghai, China | DMSO | 100 µg/mL at -20 ℃ |
| **18** | Voriconazole | Aladdin, Shanghai, China | DMSO | 6.4 mg/mL at -20℃ |
| **19** | Itraconazole | Aladdin, Shanghai, China | DMSO | 6.4 mg/mL at -20℃ |
| **20** | Miconazole | Aladdin, Shanghai, China | DMSO | 6.4 mg/mL at -20℃ |
| **21** | Rhodamine 6G | Sangon biotech, Shanghai, China | DMSO | 10 mM at -20℃ |
| **22** | CaCl_2_ | Sangon biotech, Shanghai, China | H_2_O | 100 mM -20℃ |
| **23** | EGTA | Aladdin, Shanghai, China | DMSO | 10 mM at -20℃ |
| **24** | Rapamycin | Aladdin, Shanghai, China | DMSO | 10 mg/mL at -20℃ |
| **25** | Doxycycline | Aladdin, Shanghai, China | DMSO | 50 mg/mL at -20℃ |
| **26** | Fluvastatin | Aladdin, Shanghai, China | DMSO | 6.4 mg/mL at -20℃ |
| **27** | Terbinafine | Aladdin, Shanghai, China | DMSO | 6.4 mg/mL at -20℃ |
| **28** | Cholesterol | Sangon biotech, Shanghai, China | n-Hexane | 5 mg/mL at -20℃ |
| **29** | Phenazine methosulfate | Aladdin, Shanghai, China | H_2_O | 0.32 mg/mL at -20℃ |
| **30** | NaOH | Sangon biotech, Shanghai, China | Absolute ethanol | 15% Ready to use |
| **31** | Absolute ethanol | Sangon biotech, Shanghai, China | / | >99.0% at room temperature |
| **32** | MOPS | Sangon biotech, Shanghai, China | H_2_O | 0.165 M at -4℃ |
| **33** | CCK-8 | Target Molecule Corp, Shanghai,China | / | at -20℃ |
| **34** | n-hexane | Sangon biotech, Shanghai, China | / | >99.5% at room temperature |
| **35** | Petroleum ether | HuShi, Shanghai, China | / | >99.0% at room temperature |
| **36** | DMSO | Sangon biotech, Shanghai, China | / | >99.0% at room temperature |
| **37** | RPMI 1640 | Gibco, America | H_2_O | at -4℃ |
| **38** | G418 | Sigma, America | H_2_O | 200 mg/mL at -4℃ |
| **39** | 0105620117 | Otava Ltd, Ukraine | DMSO | 6.4 mg/mL at -20℃ |
| **40** | 1683-5136 | ChemDivInc, America | DMSO | 6.4 mg/mL at -20℃ |
| **41** | AO-854/43467814 | Specs, Netherlands | DMSO | 6.4 mg/mL at -20℃ |
| **42** | 0105850737 | Otava Ltd, Ukraine | DMSO | 6.4 mg/mL at -20℃ |
| **43** | 1682-7428 | ChemDivInc, America | DMSO | 6.4 mg/mL at -20℃ |
| **44** | AN-329/43385639 | Specs, Netherlands | DMSO | 6.4 mg/mL at -20℃ |
| **45** | AO-854/43467938 | Specs, Netherlands | DMSO | 6.4 mg/mL at -20℃ |
| **46** | 6049471 | ChemBridge Corporation, America | DMSO | 6.4 mg/mL at -20℃ |
| **47** | 1683-5120 | ChemDivInc, America | DMSO | 6.4 mg/mL at -20℃ |
| **48** | 9038323 | ChemBridge Corporation, America | DMSO | 6.4 mg/mL at -20℃ |
| **49** | 2159-1361 | ChemDivInc, America | DMSO | 6.4 mg/mL at -20℃ |
| **50** | AO-854/43467845 | Specs, Netherlands | DMSO | 6.4 mg/mL at -20℃ |
| **51** | phosphatidylcholine | Target Molecule Corp, Shanghai,China | DMSO | 5 mg/mL at -20℃ |
| **52** | phosphatidylethanolamine | Aladdin, Shanghai, China | DMSO | 5 mg/mL at -20℃ |
| **53** | ER-tracker Red | MCE, Shanghai, China | DMSO | 5 mg/mL at -20℃ |
| **54** | DiBAC4(3) | MCE, Shanghai, China | DMSO | 10 mM at -20℃ |

[1] Uribe CC, Dos Santos de Oliveira F, Grossmann B, et al. Cytotoxic effect of amphotericin b in a myofibroblast cell line. Toxicol In Vitro, 2013, 27: 2105-2109

[2] Noble SM, Johnson AD. Strains and strategies for large-scale gene deletion studies of the diploid human fungal pathogen candida albicans. Eukaryot Cell, 2005, 4: 298-309

[3] Chang P, Wang W, Igarashi Y, et al. Efficient vector systems for economical and rapid epitope-tagging and overexpression in candida albicans. J Microbiol Methods, 2018, 149: 14-19

[4] O'Boyle NM, Banck M, James CA, et al. Open babel: An open chemical toolbox. J Cheminform, 2011, 3: 33

[5] Eberhardt J, Santos-Martins D, Tillack AF, Forli S. Autodock vina 1.2.0: New docking methods, expanded force field, and python bindings. J Chem Inf Model, 2021, 61: 3891-3898

[6] Trott O, Olson AJ. Autodock vina: Improving the speed and accuracy of docking with a new scoring function, efficient optimization, and multithreading. J Comput Chem, 2010, 31: 455-461

[7] Bowers KJC, D. E.; Xu, H.; Dror, R. O.; Eastwood, M. P.; Gregersen, B. A.; Klepeis, J. L.; Kolossvary, I.; Moraes, M. A.; Sacerdoti, F. D.; Salmon, J. K.; Shan, Y.; Shaw, D. E. Molecular dynamics simulations on commodity clusters. Proceedings of the 2006 ACM/IEEE Conference on Supercomputing, 2006, pp 84−97.

[8] Shaw DE. Desmond molecular dynamics system.

[9] Lu H, Li W, Whiteway M, et al. A small molecule inhibitor of erg251 makes fluconazole fungicidal by inhibiting the synthesis of the 14alpha-methylsterols. mBio, 2023, 14: e0263922

[10] Yang Y, Wang C, Zhuge Y, et al. Photodynamic antifungal activity of hypocrellin a against candida albicans. Front Microbiol, 2019, 10: 1810
